# Interpretable and explainable artificial intelligence for wearable sensor-based fall risk assessment in older adults: a systematic review with considerations for prosthetics and orthotics

**DOI:** 10.3389/fncom.2026.1860978

**Published:** 2026-07-14

**Authors:** Adeel Hussain Samdani, Saad Jawaid Khan, Muhannad Farhan, Kehinde Quasim Yusuf, Abu Zeeshan Bari, Muhammad Faisal Siddiqui, Usaimah Nasir, Naveed Ahmed, Saleh Ahmed Alqahtani, Yousef Mohammed Saad Alshahrani

**Affiliations:** 1 Department of Computer Engineering, Sir Syed University of Engineering and Technology, Karachi, Pakistan; 2 Department of Prosthetics and Orthotics, College of Medical Rehabilitation Sciences, Taibah University, Al Madinah Al Munawwarah, Saudi Arabia; 3 Department of Engineering Technology, Sam Houston State University, Huntsville, TX, United States; 4 Department of Computer Engineering, College of Computer Science and Information Technology, King Faisal University, Al Ahsa, Saudi Arabia; 5 Faculty of Information Technology and Electrical Engineering, University of Oulu, Oulu, Finland; 6 School of Allied Health Professions and Pharmacy, Keele University, Keele, United Kingdom

**Keywords:** explainable artificial intelligence, fall risk assessment, interpretable machine learning, older adults, prosthetics and orthotics, translational research, wearable sensors

## Abstract

**Background:**

Falls among older adults are a leading cause of morbidity and loss of independence. Wearable sensors combined with machine learning (ML) offer opportunities for objective fall risk evaluation, but low model transparency limits clinical adoption. Interpretable and explainable artificial intelligence (XAI) methods can address this constraint, yet their application in wearable sensor–based fall risk assessment has not been systematically examined.

**Methods:**

A PRISMA 2020–compliant systematic review was conducted across PubMed, Scopus, Web of Science, and IEEE Xplore. Studies were eligible if they included older adults, employed wearable sensors, hybrid sensor systems, or structured clinical assessment instruments, applied AI/ML for fall risk assessment (not detection), and incorporated intrinsic interpretability or *post-hoc* XAI. Data were extracted on population characteristics, sensor modalities, outcome definitions, ML algorithms, explainability strategies, validation methods, and predictive performance.

**Results:**

Eleven studies (2019–2025, total *n* = 5,484) met inclusion criteria. Inertial Measurement Units predominated. Fall risk definitions were heterogeneous, spanning retrospective fall history, clinical balance scales, and prospective diaries. Explainability was implemented almost exclusively at the global level, with only two studies providing both global and local explanations. On comparable tasks, interpretable models achieved accuracy of 0.65–0.92 and AUC of 0.70–0.92, suggesting interpretability carried no consistent performance penalty over more complex designs. All studies relied on internal validation only; none performed external validation or real-time deployment. None recruited P&O users or incorporated device-specific predictors.

**Conclusion:**

Current models favour interpretable architectures and achieve moderate-to-high performance, but are constrained by heterogeneous outcome definitions, absence of external validation, and global-only explainability that limits individual-level clinical utility. The evidence base does not yet support clinical deployment. Extending these findings to prosthetics and orthotics users, a clinically important downstream application, will require device-specific datasets, asymmetry-adjusted thresholds, and instance-level explanations. These represent the priority directions for the next stage of this research agenda.

## 1 Introduction

Falls among older adults represent a significant and growing public health problem, contributing to morbidity, mortality, loss of independence, and substantial healthcare costs worldwide. Approximately one in three community-dwelling adults aged 65 years and over fall at least once a year, and many sustain injuries requiring medical attention or hospitalisation (Rubenstein, 2006; Gale et al., 2016; Chen et al., 2021). Beyond the immediate physical consequences, falls are associated with long-term functional decline, fear of falling, and increased institutionalisation (Xiong et al., 2024; Li et al., 2023; Alqahtani and Alenazi, 2025). As the global population ages, the need for effective, scalable, and clinically actionable fall risk assessment has become urgent. This need is further amplified among older adults with mobility impairments, particularly users of lower-limb prostheses and orthoses, who face additional challenges arising from altered gait mechanics, balance compensation strategies, and device-specific instability (Finco et al., 2023).

Current clinical approaches to fall risk assessment rely on standardised instruments such as the Berg Balance Scale, Timed Up and Go test, and performance-oriented mobility assessments (Omaña et al., 2021; Montero-Odasso et al., 2022; Beck Jepsen et al., 2022). Although widely used, these tools are constrained by subjectivity, ceiling effects, and limited predictive validity, with reported AUC values of approximately 0.5–0.7 in prospective fall prediction (Beck Jepsen et al., 2022; Jepsen et al., 2022; Kozinc et al., 2020; Meekes et al., 2021). A further limitation is that the assessments are conducted at discrete time points in controlled environments, which may not capture the dynamic and context-dependent mobility patterns that precede falls in real-life scenarios. For prosthesis and orthosis users, this limitation is especially consequential. Device-specific gait adaptations, alignment-related instability, and fatigue-related compensatory strategies emerge during real-world ambulation for prosthesis and orthosis users and are largely invisible to clinic-based assessment.

Physical functional limitations represent a primary predisposing factor for falls: reduced gait speed, impaired balance, diminished lower extremity strength, and altered spatiotemporal gait parameters are consistently associated with both frailty and fall risk in community-dwelling older adults (Ruiz-Ruiz et al., 2021; Bezold et al., 2021). Wearable inertial sensor studies have demonstrated that these gait disruptions can be objectively captured and used to discriminate between fallers and non-fallers, with trunk stability parameters emerging as particularly sensitive indicators (Ruiz-Ruiz et al., 2021; Bezold et al., 2021). Beyond biomechanical factors, cognitive impairment substantially compounds fall risk, and accelerometer-derived physical activity patterns, including reduced activity intensity, diminished movement variability, and disrupted circadian rhythmicity, have been shown to reflect cognitive status and its decline over time (Shi et al., 2022; Fan L. J. et al., 2025). Physical function impairment and cognitive decline are themselves interrelated, with wrist accelerometer digital biomarkers capturing activity transition patterns and frequency-domain features that correspond to objective physical performance measures (Fan et al., 2024; Fan L. et al., 2025). Chronic pain adds a further layer of vulnerability: site-specific pain, particularly in the back, neck, and lower limbs, is associated with reduced total activity, increased sedentary time, and altered activity fragmentation patterns that may disrupt the movement regularity underpinning postural stability (Fan L. et al., 2025). Collectively, these converging physical, cognitive, and pain-related pathways underscore the multifactorial nature of fall risk and the limitations of any assessment approach that captures only one dimension at a single time point.

Wearable sensor technologies, including inertial measurement units, pressure sensors, and multimodal physiological devices, offer a means of addressing these limitations by enabling objective, continuous monitoring in both laboratory and free-living settings (Picerno et al., 2021; Bargiotas et al., 2023). The rich time-series data they generate can capture subtle gait and balance impairments that are consistently associated with fall risk, including reduced gait speed, increased stride time variability, and impaired trunk stability. Machine learning (ML) approaches are well-suited to modelling the complex, multifactorial nature of this data, and a growing body of work has demonstrated that sensor-based ML models can predict fall risk with discrimination performance that meaningfully exceeds that of traditional clinical screening tools (Lin et al., 2025; Zhou et al., 2025). For prosthetics and orthotics practice specifically, the combination of wearable sensors and interpretable ML represents a pathway toward objective, device-aware fall risk assessment. This is an area not currently addressed by clinical tools or existing evidence syntheses.

A critical requirement for clinical adoption, however, is that the model outputs must be interpretable by clinicians. In fall risk assessment, this means that a clinician must be able to understand which sensor-derived features contribute to an individual’s risk estimate, and why, so that the output can inform rehabilitation planning, component selection, or referral decisions rather than functioning as a black box. This need for interpretability is reinforced by emerging regulatory frameworks, including the European Union’s Artificial Intelligence Act and guidance from the U. S. Food and Drug Administration, both of which require that AI systems used in medical contexts be transparent, explainable, and subject to meaningful clinical oversight (Smuha, 2025; Yadav, 2025). Despite growing recognition of this requirement, interpretability and explainability have not yet been examined as central analytical dimensions in the wearable sensor-based fall risk literature.

Several reviews have examined related topics, but none addresses this intersection directly. Earlier and recent reviews of sensor-based fall risk assessment have identified methodological challenges relating to outcome definitions and generalisability (Kristoffersson et al., 2021; Montesinos et al., 2018). Reviews of ML approaches to fall prediction have typically incorporated heterogeneous data sources well beyond wearable sensors (Bargiotas et al., 2023; Yu et al., 2025). Reviews addressing explainable AI in healthcare have done so in general methodological terms, without reference to the fall risk domain or wearable sensor data specifically (Band et al., 2025; Osonuga et al., 2026). A substantial parallel literature addresses fall *detection* systems, but this is concerned with algorithmic performance and sensing modalities for acute-event recognition rather than prospective risk assessment (Nooruddin et al., 2022; Akter et al., 2025). Critically, to the best of our knowledge, no existing review examines wearable sensor-based fall risk assessment through the lens of interpretable and explainable AI, and none addresses the translational relevance of this evidence for prosthetics and orthotics practice.

The aim of this systematic review is therefore to critically synthesise the literature on interpretable and explainable AI methods for fall risk assessment in older adults using wearable sensors, and to evaluate the translational relevance of this evidence for prosthetics and orthotics practice. Specifically, the objectives are to: (i) describe the wearable sensor modalities, tasks, and outcomes investigated; (ii) summarise the AI and explainability methods used; (iii) evaluate model performance and validation approaches; (iv) assess the extent to which explainability supports clinical interpretation and real-world applicability; and (v) identify the evidence gaps that must be addressed before this evidence base can be extended to prosthesis and orthosis users.

## 2 Methods

### 2.1 Study selection and search strategy

This systematic review was not prospectively registered in PROSPERO or another systematic-review registry. The methodology was developed *a priori* and conducted in accordance with PRISMA 2020 guidelines. This absence of pre-registration is acknowledged as a limitation.

The research question and eligibility criteria for this systematic review were developed according to the PICOS framework. The population (P) comprised older adults undergoing fall-risk assessment. The intervention/exposure (I) included wearable sensors, hybrid sensor systems, and structured clinical assessment instruments used in conjunction with AI or ML approaches. Comparator groups (C), where applicable, included fallers versus non-fallers or different levels of fall risk. Outcomes (O) included fall-risk prediction, classification, stratification, or phenotyping performance, along with explainability and interpretability characteristics of the models. Eligible study designs (S) included observational and predictive modelling studies incorporating interpretable or explainable AI methods.

This systematic review was performed based on the Preferred Reporting Items of Systematic Review and Meta-Analysis (PRISMA) guideline (Tugwell and Tovey, 2021). The extensive literature search was conducted between December 2025 and January 2026, in large electronic databases such as PubMed, Scopus, Web of science, and IEEE Xplore since their inception till the latest search date. The search strategy (see Supplementary Table S1 for full search strings) was a combination of controlled vocabulary and free-text search terms that were related to: (i) falls and fall risk (e.g., fall, fall risk, fall prediction), (ii) wearable and hybrid sensing technologies (e.g., wearable sensor, IMU, accelerometer, gyroscope, pressure sensor), (iii) artificial intelligence and machine learning (e.g., machine learning, deep learning, classification), and (iv) interpretability and explainability (e.g., interpretable, explainable AI, XAI, decision tree, rules, feature importance). Bibliometric analyses of the included studies were conducted via online tools such as Connected Papers (Behera et al., 2023) and SciSpace (Jain et al., 2024) by inputting the core papers. These tools were used as supplementary reference-chaining aids to identify potentially missed studies. After duplicate removal, two reviewers independently screened titles and abstracts. Potentially relevant full-text articles were subsequently retrieved and independently assessed by the same two reviewers for eligibility. Any disagreements at either stage were resolved through discussion and, when necessary, consultation with a third reviewer. Data extraction was performed by two reviewers and checked by a third reviewer in case of conflicts.

A complete PRISMA 2020 checklist can be found as Supplementary Table S2.

### 2.2 Eligibility criteria

For eligibility, the studies had to satisfy the following criteria:

- Included older adults (mean age ≥50 years or literally defined as older populations)
- Used wearable sensors, hybrid sensor systems, or structured clinical assessment instruments to gather kinematic, physiological, postural, or functional information.
- Fall risk assessment, faller classification or fall-related phenotyping by using applied artificial intelligence or machine learning techniques.
- Models and techniques of intrinsic interpretability or *post-hoc* explainability are reported.
- Quantitative measures of performance of the model are reported.
- Were published in English as peer-reviewed journal articles.

The studies were filtered out if they were conference proceedings, focused solely on fall detection without risk assessment, used non-human or simulated datasets only, did not implement AI-based modelling, or lacked any interpretable or explainable component.

### 2.3 Definitions of interpretable and explainable artificial intelligence

For the purposes of this review, the concept of interpretable AI was modelling approaches whose internal structure and decision logic are inherently transparent and directly understandable without auxiliary explanation tools. They include white-box models, which are logistic regression, decision trees, rule-based systems, and fuzzy logic models, with the feature contributions or decision paths being explicitly traceable.

Explainable AI (XAI) was described as the use of *post-hoc* or hybrid methods that can give human-understandable explanations of the predictions made by otherwise complex or black box models. Common *post-hoc* techniques include feature-attribution methods such as SHAP and LIME, and surrogate explanation models. For the purposes of this review, models were categorised as: (i) intrinsically interpretable, models whose decision logic is directly readable from their structure (e.g., Decision Trees, Logistic Regression, Elastic Net); (ii) black-box with *post-hoc* explainability, opaque models (e.g., Gradient-Boosted Ensembles, Deep Neural Networks) to which external explanation methods were applied after training; or (iii) hybrid models that incorporate both opaque learning components and architectural mechanisms that generate interpretable outputs as part of the model itself, such as self-attention weight maps.

### 2.4 Data extraction

A template was created in Microsoft Excel as a standardized extraction template. The variables that were extracted were categorised into six domains, which included (1) sample and clinical characteristics, (2) sensors and data acquisition, (3) task and outcome definitions, (4) AI/ML model characteristics, (5) explainability methods, and (6) validation and performance outcomes.

### 2.5 Explainability indicators and categorization

The included models were categorised as interpretable, explainable, or hybrid based on their transparency characteristics and the use of *post-hoc* explanation techniques. Interpretable models referred to inherently transparent models in which the decision-making process could be directly understood from the model structure, whereas explainable models relied on *post-hoc* XAI techniques to generate explanations for model predictions. Hybrid models combined interpretable model structures with additional explainability methods.

The explanation outputs were further classified according to their scope as global explanations (model-level explanations describing overall model behaviour and feature importance), local explanations (instance-level explanations describing the contribution of features to individual predictions), or both. Studies were examined for the presence of clinically meaningful explanatory variables, including measures such as gait speed, balance, mobility, functional performance, and other clinically interpretable fall risk indicators.

### 2.6 Data synthesis

Quantitative meta-analysis was not possible as the heterogeneity of sensors, outcome definitions, modelling approaches and validation processes was substantial. The synthesis of qualitative data was thus carried out in a systematic manner. The studies were clustered based on sensor modality, modelling paradigm and explainability method. The sample sizes were summarized by descriptive statistics to give the distributions of sample sizes, sensors used, model types, and methods of XAI. The visualization of data was conducted with the help of Microsoft Excel (Microsoft Corporation, Redmond, WA, USA) and Flourish Studio (Flourish, London, UK).

### 2.7 Risk of bias assessment

A slightly modified version of the Prediction model Risk of Bias Assessment Tool (PROBAST) (Moons et al., 2025) was used to determine the methodological quality of the included studies. Due to pervasive gaps in methodological reporting across the included literature, the assessment was restricted to three of the four PROBAST domains that could be evaluated with confidence from the available information: Participants, Outcomes, and Analysis. The Predictors domain was excluded because while predictor definitions were generally identifiable across studies, the measurement and feature extraction approaches were inconsistently documented.

As a result, the overall bias ratings reported here may not capture the full spectrum of methodological risk, and this represents a limitation of the review. Risk of bias assessment for each included study (low, high, or unclear) was conducted independently by two reviewers (AH and SJK). Any disagreements were resolved through discussion and, when necessary, consultation with a third reviewer (AZB).

### 2.8 Quality of evidence

The TRIPOD-AI (Transparent Reporting of a multivariable prediction model for Individual Prognosis or Diagnosis-Artificial Intelligence) guidelines were used to determine the quality of evidence (de Kanter et al., 2025). All studies were assessed in the areas of TRIPOD-AI reporting that were related, such as data sources, selection of participants, definition of outcome, model development, the strategy of validation, and reporting transparency. Conformance to personal TRIPOD-AI items was also noted as adequately reported, partially reported, or not reported. General evidence quality was then described in a descriptive manner to determine general strengths and weaknesses in reporting and methodological rigor of the included studies.

## 3 Results

### 3.1 Study selection

**Figure 1:**
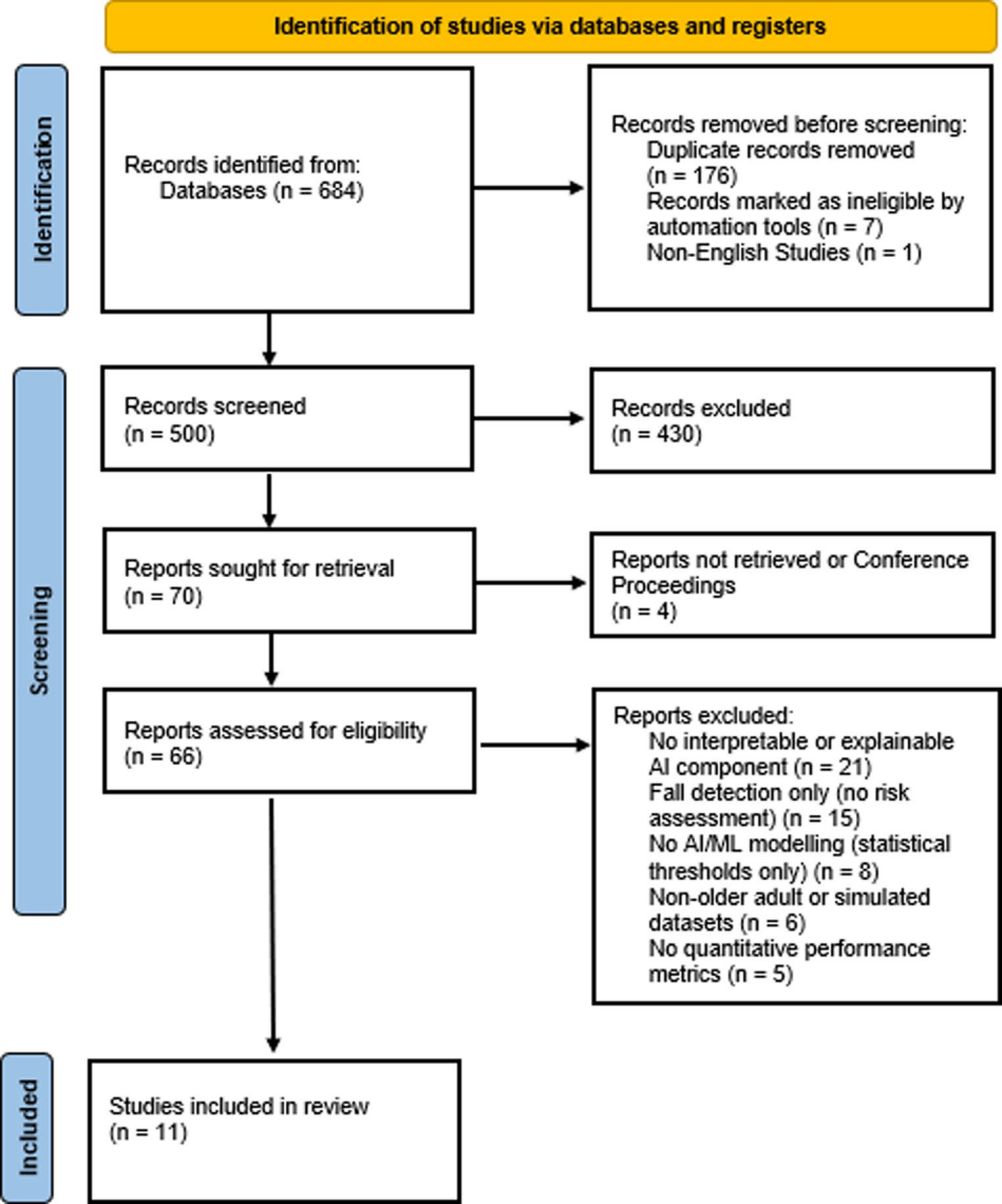
PRISMA 2020 flow diagram summarizing the study selection process, from database identification to final inclusion of eligible studies.

Database search provided 684 records. Following a deletion of duplicates and other ineligible records, 500 articles were then screened by title and abstract leading to 430 being excluded. A total of 70 full-text reports were sought to be retrieved, and 66 of them were evaluated for eligibility. After going through the entire text, 55 articles were eliminated, mainly because either they did not contain any interpretable or explainable AI elements, were interested in fall detection, did not have AI/ML modelling, were not based on older adults, had simulation datasets, or did not provide the quantitative performance measures. A total of 11 studies passed the inclusion criteria and were incorporated in the final review (Figure 1). Supplementary Table S3 provides the summary table of the included studies.

### 3.2 Study characteristics

Studies originated from (based on the country of the first author): Asia [Japan (Sato and Watanabe, 2025; Makino et al., 2021), China (Wang et al., 2024), Republic of Korea (Noh et al., 2021), Taiwan (Liang et al., 2024), Hong Kong SAR (Fong et al., 2023)], Europe [France (El Marhraoui et al., 2023), Belgium (Gillain et al., 2019)], North America (USA) (Mishra et al., 2022), and Oceania [New Zealand (Mohan et al., 2025)]. The included studies were published between 2019 and 2025. The largest number of publications was in 2021 (*n* = 3) (Makino et al., 2021; Noh et al., 2021; Schniepp et al., 2021).

### 3.3 Population and clinical context

**Figure 2:**
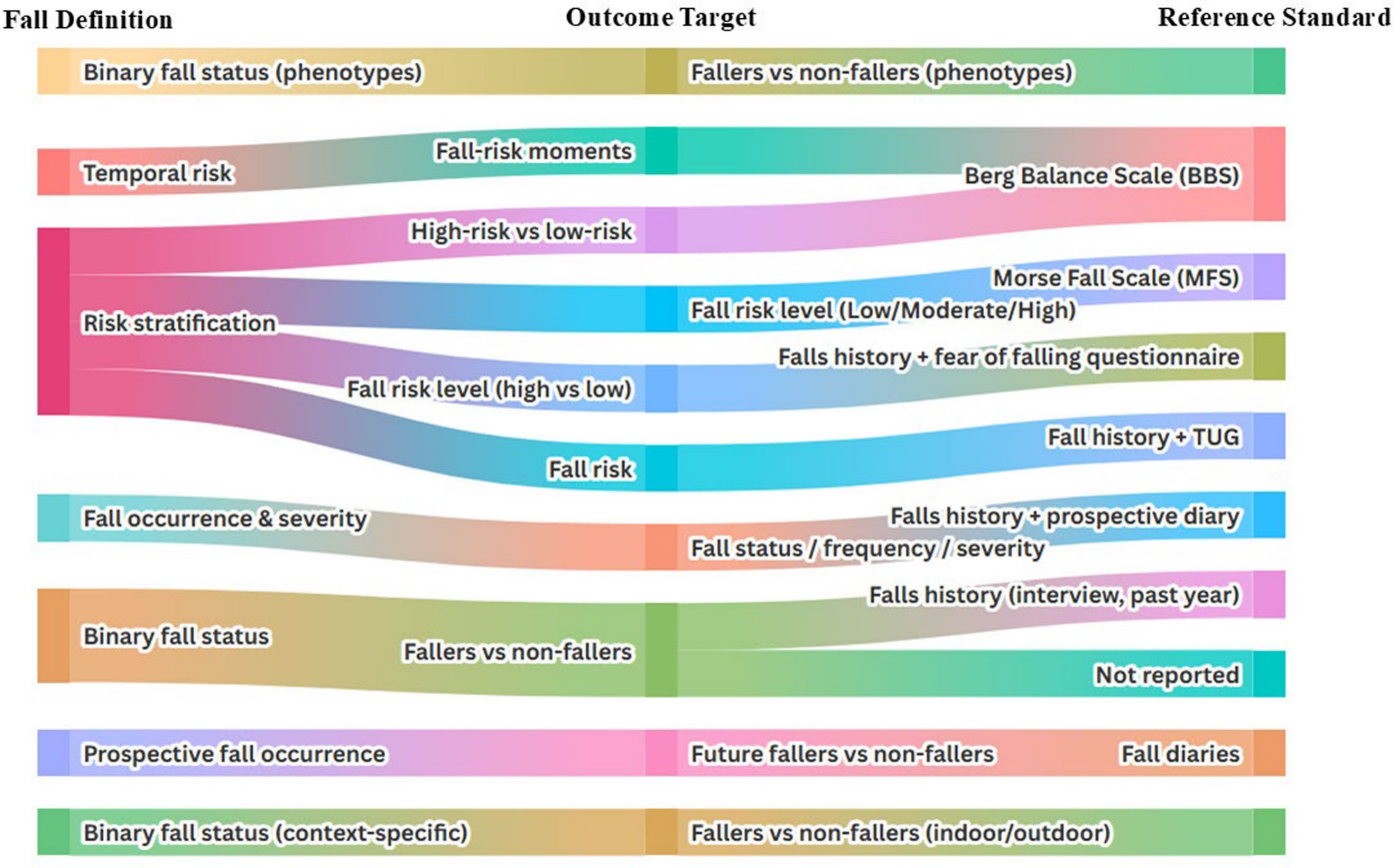
Alluvial diagram illustrating the relationships between fall definitions, outcome targets, and reference standards used across included studies of interpretable and explainable AI for fall risk assessment in older adults. Each flow represents one study.

The variation in number of participants among different studies was vast (27–2,520) (Figure 2a). Nonetheless, explicit training and testing partitions were only reported in four studies with training sets of between 30 and 810 participants and test sets of between 15 and 341 participants.

The mean age of the participants across the studies was between about 53 and 86 years with majority of the cohorts having adult population aged above 70 years. Various reports considered community-based older adults, whereas other articles incorporated mixed or neurological samples that had broader age ranges. In total, 5,484 participants were investigated. Among these, 61% of them were obtained by the studies that included exclusively healthy community-dwelling older adults; 38% of them were obtained from studies reporting mixed populations, including frail and comorbid older adults, as well as cohorts that explicitly reported engaging in neurological conditions, e.g., stroke and neurological gait disorders. Notably, disaggregated populations of subgroups were not reported in most mixed-population studies, which impeded specific quantification of condition-specific sample sizes. Regarding the recruitment setting, six studies were performed in older adults that live in the community (Makino et al., 2021; Wang et al., 2024; Noh et al., 2021; Liang et al., 2024; Fong et al., 2023; Gillain et al., 2019).

### 3.4 Fall and outcome targets definitions

Heterogeneous criteria were used to define falls and fall risk in studies (falls history, clinical balance scales (BBS, TUG, MFS), and prospective fall diaries). The definitions of falls were separated into conceptual definitions to be visualized. Figure 2 presents a Sankey diagram illustrating the relationships between fall definitions, outcome targets, and reference standards used across included studies.

Reference standards fell into three categories. Retrospective fall history was the most common, used in six studies (Makino et al., 2021; Noh et al., 2021; Liang et al., 2024; Fong et al., 2023; Gillain et al., 2019; Schniepp et al., 2021), typically dichotomising participants as fallers or non-fallers based on one or more falls within a 6–12-month recall period. Clinical balance and mobility scales served as proxies in three studies: the Berg Balance Scale (BBS ≤ 50 indicating high risk) (Wang et al., 2024), the TUG test alone or combined with fall history (≥10 s threshold) (Liang et al., 2024), and the Morse Fall Scale stratified into low, moderate, and high-risk categories (Mohan et al., 2025). Prospective monitoring was used in two studies, with follow-up periods of 6 months (Schniepp et al., 2021) and 2 years (Gillain et al., 2019) respectively, enabling outcome definitions based on observed rather than recalled fall occurrence.

### 3.5 Fall risk determinants identified by interpretable/explainable AI models

A key contribution of applying interpretable and explainable AI (XAI) in fall risk research is the ability to articulate which features drive model decisions. Across the 11 studies reviewed, explainability outputs, whether intrinsic (decision-tree rules, elastic net coefficients, fuzzy rules) or *post-hoc* (SHAP values, attention weights, gain-based feature importance), consistently highlighted a convergent set of fall risk determinants. Table 1 below consolidates these features, grouped by domain, and maps each to the studies that identified it and its clinical significance.

**Table 1:** Fall risk determinants identified by interpretable/explainable AI model, grouped by feature domains.

Feature domain	Feature/risk factor	Studies identifying feature	Clinical relevance and XAI insight
Temporal Gait	Walking speed/gait speed	Sato and Watanabe (2025), Noh et al. (2021), Fong et al. (2023), and Gillain et al. (2019)	Slower speed linked to fall risk; consistent with clinical gait assessment standards
Temporal Gait	Cadence/step frequency	Sato and Watanabe (2025) and Noh et al. (2021)	Reduced cadence reflects compensatory gait in fear-of-fall phenotypes
Temporal Gait	Stride time variability/regularity	Noh et al. (2021), Liang et al. (2024), and Gillain et al. (2019)	Greater variability is a marker of impaired motor control and fall risk
Spatial Gait	Stride length/step length	Sato and Watanabe (2025), Noh et al. (2021), and Gillain et al. (2019)	Shortened stride is a compensatory strategy; strongly associated with fall occurrence
Spatial Gait	Stance phase duration/double support time	Wang et al. (2024) and Noh et al. (2021)	Prolonged stance/double support reflects instability avoidance
Spatial Gait	Minimum toe clearance (MTC)	Gillain et al. (2019)	Low MTC is a direct tripping hazard; difficult to capture without lab instrumentation
Balance/Posturography	Postural sway features (COP-based)	Liang et al. (2024), El Marhraoui et al. (2023), and Mohan et al. (2025)	SHAP revealed sway-derived features (e.g., sway area, velocity) as top predictors; attention weights highlighted dynamic balance-loss moments
Balance/Posturography	Acceleration magnitude/jerk (trunk/ankle)	Wang et al. (2024), El Marhraoui et al. (2023), and Schniepp et al. (2021)	Trunk acceleration indices capture instability during locomotion; ankle jerk peaks associated with near-fall events
Balance/Posturography	Gait symmetry/regularity indices	Sato and Watanabe (2025) and Gillain et al. (2019)	Asymmetry is a hallmark of neurological gait disorders associated with falls
Clinical/Functional	Fall history (retrospective)	Sato and Watanabe (2025), Makino et al. (2021), Fong et al. (2023), Schniepp et al. (2021)	Strongest single predictor in logistic regression (Schniepp et al., 2021); consistent with clinical consensus
Clinical/Functional	Timed Up and Go (TUG) score	Makino et al. (2021), Wang et al. (2024), Liang et al. (2024), Fong et al. (2023)	TUG ≥ 9.7–18 s identified as key threshold across decision-tree models; robust clinical benchmark
Clinical/Functional	Fear of falling (FES-I/FOF questionnaire)	Sato and Watanabe (2025) and Noh et al. (2021)	Interacts with gait phenotype (SHAP), High FOF predicts cautious gait pattern with elevated fall risk
Clinical/Functional	Functional Reach/Berg Balance Scale	Wang et al. (2024), Fong et al. (2023), and Gillain et al. (2019)	BBS and Fall Risk used as reference standards and/or input features; thresholds (BBS ≤ 50; FR < 15–17 cm) align with clinical cut-offs
Clinical/Functional	Frailty/physical function indices (SPPB, Fried)	Sato and Watanabe (2025), Makino et al. (2021), and Mishra et al. (2022)	Phenotypic frailty modulates the relative importance of gait vs. clinical features
Clinical/Functional	Polypharmacy/number of medications	Makino et al. (2021) and Fong et al. (2023)	Decision-tree rules incorporated medication burden alongside mobility measures such as TUG and FR, using thresholds of ≥2 drugs (Fong et al., 2023) and ≥5 drugs/polypharmacy (Makino et al., 2021).
Clinical/Functional	BMI/anthropometrics	Fong et al. (2023)	BMI ≥ 22 appeared as a branch in the Fong decision tree for community-dwelling older adults

Temporal and spatial gait features, particularly walking speed, stride length, and stride-time variability, were the most consistently reported determinants across sensor-based studies, aligning with established biomechanical theory. Walking speed emerged in at least four studies and was identified as a top SHAP contributor in the phenotype-stratified analysis by Sato et al. (Sato and Watanabe, 2025), where its relative importance varied by gait phenotype (e.g., dominant in the ‘robust’ phenotype but secondary to fear-of-falling in the ‘cautious’ phenotype). Noh et al. (Noh et al., 2021) demonstrated that stride length and stance phase duration maintained predictive importance across multiple self-selected walking speeds, highlighting their robustness as features.

Balance and postural control features were prominently identified in studies employing postural assessment paradigms (Liang et al., 2024; El Marhraoui et al., 2023) and instrumented TUG protocols (Wang et al., 2024). The self-attention mechanism employed by El Marhraoui et al. (2023) uniquely highlighted specific temporal windows of elevated instability during exercise tasks in stroke survivors, offering a form of local, instance-level explainability not captured by global feature rankings alone.

Among clinical and functional predictors, fall history was the single strongest predictor in regression-based studies (Schniepp et al., 2021) and appeared as a primary branching criterion in multiple decision trees (Makino et al., 2021; Fong et al., 2023). The TUG test, used both as a reference standard and as a feature, yielded clinically interpretable thresholds (≥ 9.7–18 s) across four studies (Makino et al., 2021; Wang et al., 2024; Liang et al., 2024; Fong et al., 2023), and its segmentation into sub-tasks by Wang et al. (Wang et al., 2024) revealed that specific movement phases (e.g., turning) contribute disproportionately to fall risk. Fear of falling, when included, showed phenotype-dependent importance: critical in cautious walkers (Sato and Watanabe, 2025) but less prominent in robust or high-cadence profiles.

Taken together, XAI outputs across the reviewed studies confirm that fall risk is multifactorial and domain-specific: no single feature universally dominates. The convergence on walking speed, fall history, TUG performance, and stride length provides a data-driven corroboration of clinically established risk factors, while phenotype-stratified SHAP analysis introduces nuance, suggesting that the relative importance of features is population- and context-dependent. Future XAI-driven research should explicitly aim to characterise these interactions to better support individualised fall risk assessment.

### 3.6 Sensor modalities and data acquisition

**Figure 3:**
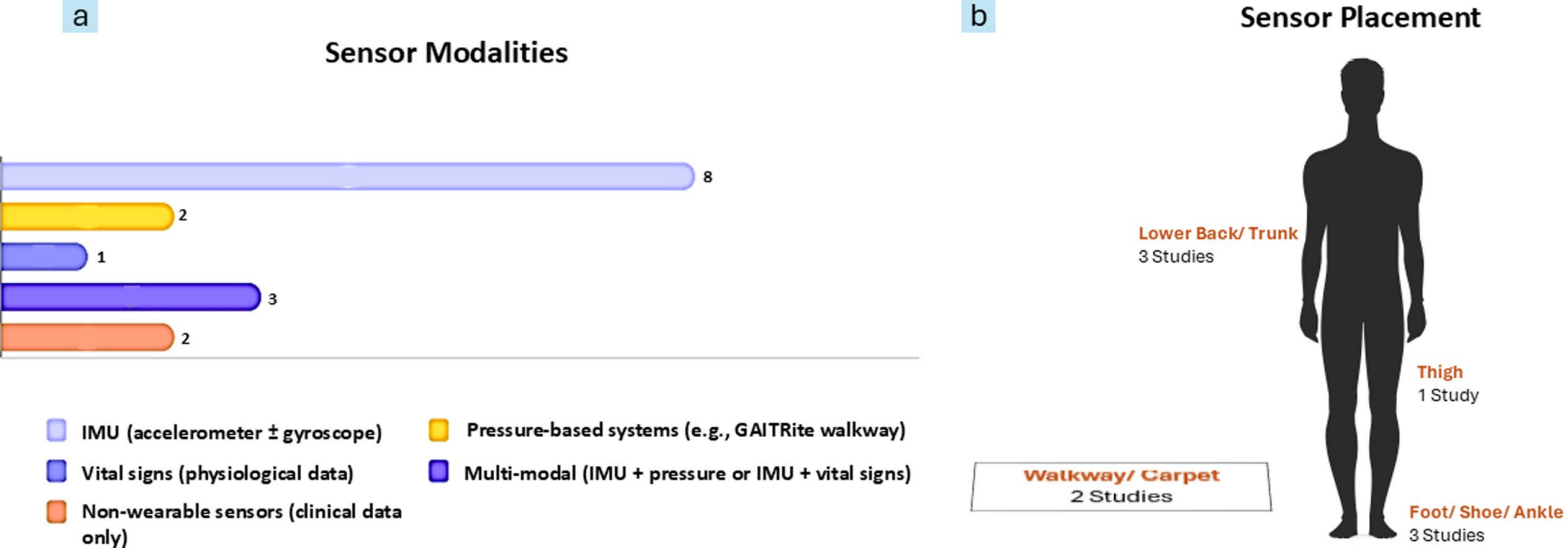
Overview of sensing strategies employed in included studies. **(a)** Distribution of sensor modalities, **(b)** anatomical distribution of wearable sensor placement.

IMU-based sensing was the dominant modality, employed across seven studies using accelerometers with or without gyroscopes (Sato and Watanabe, 2025; Wang et al., 2024; Noh et al., 2021; Liang et al., 2024; El Marhraoui et al., 2023; Gillain et al., 2019; Schniepp et al., 2021) (see Figure 3a). Pressure-based systems, including instrumented walkways, were used in two studies (Mishra et al., 2022; Schniepp et al., 2021), and one study relied on physiological vital signs, where AI2 component used an accelerometer-based ADL dataset (Mohan et al., 2025). Two studies used no wearable sensors at all, relying exclusively on structured clinical assessments such as fall history interviews, functional test scores, and questionnaires (Makino et al., 2021; Fong et al., 2023). These were retained under the broadened eligibility criterion permitting clinical assessment instruments as a data source.

Sensor placement varied: the lower back or trunk (Wang et al., 2024; Liang et al., 2024; Gillain et al., 2019), thigh (Schniepp et al., 2021), and the foot, shoe, or ankle (Sato and Watanabe, 2025; Noh et al., 2021; El Marhraoui et al., 2023) (see Figure 3a). Three studies did not report sensor placement (Makino et al., 2021; Fong et al., 2023; Mohan et al., 2025). Sampling frequencies ranged widely from 5 Hz to 120 Hz, with variation reflecting both sensor type and task demands (Sato and Watanabe, 2025; Wang et al., 2024; Noh et al., 2021; El Marhraoui et al., 2023; Schniepp et al., 2021). Recording durations similarly spanned a wide range: most studies used short laboratory sessions of seconds to minutes (Wang et al., 2024; Noh et al., 2021; Liang et al., 2024; El Marhraoui et al., 2023; Gillain et al., 2019), one extended to 30-min walking tests (Sato and Watanabe, 2025), another to multi-day monitoring of approximately 3 days (Mohan et al., 2025), and only one included free-living monitoring for 14 days (Schniepp et al., 2021).

### 3.7 Data collection context

Data collection settings fell into three broad categories. Most studies (*n* = 6) were conducted in controlled laboratory or clinical environments, using structured walking tests, TUG protocols, or exercise-based recordings (Sato and Watanabe, 2025; Wang et al., 2024; Noh et al., 2021; Liang et al., 2024; El Marhraoui et al., 2023; Gillain et al., 2019). A smaller group employed mixed environments combining short-term laboratory assessment with longer monitoring periods or real-life data collection (Mishra et al., 2022; Schniepp et al., 2021). Two studies were conducted entirely in community-based, non-laboratory settings, one in community centres (Liang et al., 2024) and one in primary healthcare settings without wearable devices (Fong et al., 2023).

### 3.8 Prediction task formulation

#### 3.8.1 Binary faller classification (faller vs. non-faller)

Binary classification was the most common prediction task formulation across the included studies, with six studies defining the outcome as faller versus non-faller status. Reference standards varied considerably. Retrospective fall history over a 6–12-month recall period was the most frequent approach, used across several studies that dichotomised participants based on self-reported or interview-confirmed falls (Makino et al., 2021; Noh et al., 2021; Liang et al., 2024; Fong et al., 2023; Gillain et al., 2019; Schniepp et al., 2021). Used future fallers and non-fallers as the dependent variable in a two-year follow-up, whereas (Schniepp et al., 2021) used both retrospective and prospective diary-based data to predict their fall status and frequency. Noh et al. (Noh et al., 2021) operationalised fall risk perception by asking a single question: *are you afraid of falls?* (Makino et al., 2021) anticipated fallers and non-fallers based on interviews, fall history, while (Fong et al., 2023) predicted fallers and non-fallers basing on self-reported 12-month prevalence (Liang et al., 2024). Also categorised the fall risk based on retrospective fall history plus TUG test thresholds.

#### 3.8.2 Multi level risk stratification

Three studies moved beyond binary classification to model fall risk as an ordered or multi-level categorical outcome, reflecting the clinical reality that risk is not dichotomous but exists along a continuum of severity and frequency (Mohan et al., 2025) forecasted the fall risk as three-class outcome (low, moderate, high) according to categories of Morse Fall Scale (Makino et al., 2021). Grouped the participants into various probability-based risk groups based on decision tree rules that were based on clinical predictors (Schniepp et al., 2021). Also estimated the severity of falls and the frequency of falls based on multi-class outcomes alongside binary faller status. Across these three studies, the shared methodological rationale is that ordinal or multi-class outcome structures better reflect the clinical gradient of fall risk than simple faller-versus-non-faller dichotomisation and are better suited to guiding tiered intervention decisions.

#### 3.8.3 Phenotyping-based prediction

A single study incorporated a phenotyping task along with faller classification (Sato and Watanabe, 2025). Categorised the participants into gait phenotypes (robust, high-cadence, intermediate, cautious) and further divided fallers and non-fallers into their respective phenotypes. Under this structure, the main activity was not confined to predicting risks but also identifying subgroups with different fall mechanisms. The difference between this task structure and the traditional fall prediction is the fact that the former is concerned with the subgroup discovery before the outcome is classified.

#### 3.8.4 Temporal risk detection

One study conceptualized fall risk as a continuous or temporal prediction task rather than a person-level classification (El Marhraoui et al., 2023). Predicted fall risk instants based on balance loss during movement based on continuous IMU data recorded on the ankle. The result was not faller state but time-localized high-risk states, which are reflected in elevated attention weights in sliding windows. This task is the opposite of faller classification in that it aims at detecting momentary risks level as opposed to detecting risk at the individual level.

#### 3.8.5 Prospective fall prediction

Two articles made use of longitudinal follow-up as an outcome (explicitly) to predict future falls (Gillain et al., 2019). Predicted fallers and non-fallers using gait and clinical parameters in a 2-year follow-up (Mishra et al., 2022). Predicted the fall occurrence over 6 months among institutionalized older adults.

### 3.9 ML model categories

**Figure 4:**
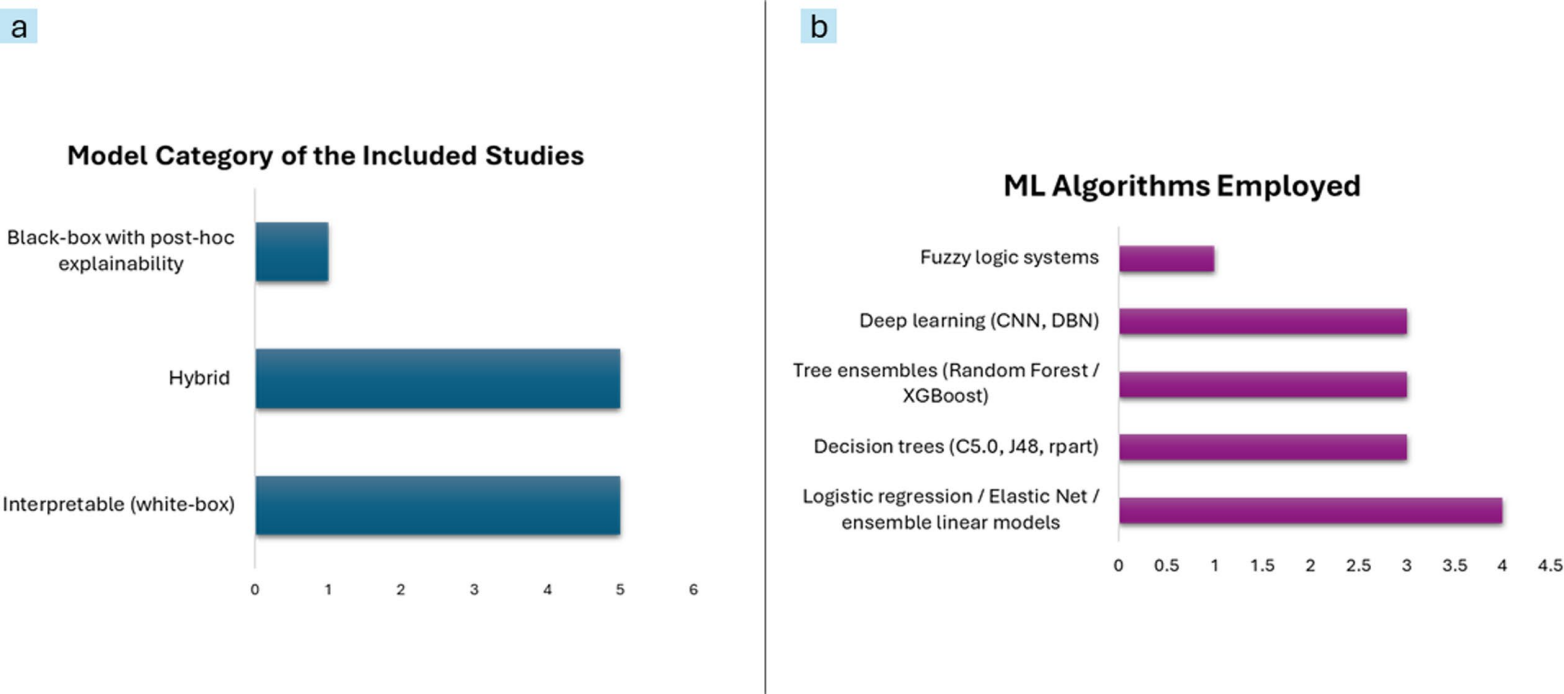
Overview of machine-learning models used across included studies. **(a)** Distribution of model categories based on interpretability (interpretable, hybrid, and black box). **(b)** Frequency of specific machine-learning algorithms employed across studies.

Five studies used interpretable models intrinsically, such as elastic net classifiers, logistic regression, and decision tree-based approaches, where model coefficients or model rules can be directly inspected (Makino et al., 2021; Wang et al., 2024; Fong et al., 2023; Gillain et al., 2019; Schniepp et al., 2021) (see Figure 4a).

Five additional studies used hybrid modelling approaches, which involved using black-box learners (e.g., neural networks or ensemble models) alongside interpretable elements to justify them (Sato and Watanabe, 2025; Liang et al., 2024; El Marhraoui et al., 2023; Mishra et al., 2022; Mohan et al., 2025). Only one study relied exclusively on a black-box model without intrinsic interpretability, using gradient-boosted trees for fall risk classification (Noh et al., 2021).

### 3.10 Algorithms and feature engineering

Traditional statistical and tree-based approaches were the most frequently used modelling strategies. Commonly applied algorithms included logistic regression-based models such as elastic net and ensemble linear classifiers, decision tree approaches including C5.0, J48, and recursive partitioning trees, and ensemble tree algorithms such as Random Forest and XGBoost (Sato and Watanabe, 2025; Makino et al., 2021; Noh et al., 2021; Fong et al., 2023; Gillain et al., 2019). Less frequently used approaches included convolutional neural networks, deep belief networks, and fuzzy logic-based hybrid systems (Sato and Watanabe, 2025; El Marhraoui et al., 2023; Mohan et al., 2025). Most studies relied on manually engineered features derived from gait signals or clinical variables, including spatiotemporal gait parameters, postural sway measures, and questionnaire scores (Sato and Watanabe, 2025; Makino et al., 2021; Wang et al., 2024; Noh et al., 2021; Liang et al., 2024; Fong et al., 2023; Gillain et al., 2019; Mohan et al., 2025; Schniepp et al., 2021), whereas El Marhraoui et al. (2023) employed an end-to-end learning approach in which features were automatically learned directly from raw IMU signals using a convolutional neural network with self-attention.

### 3.11 Level of explanation and clinical interpretation

**Figure 5:**
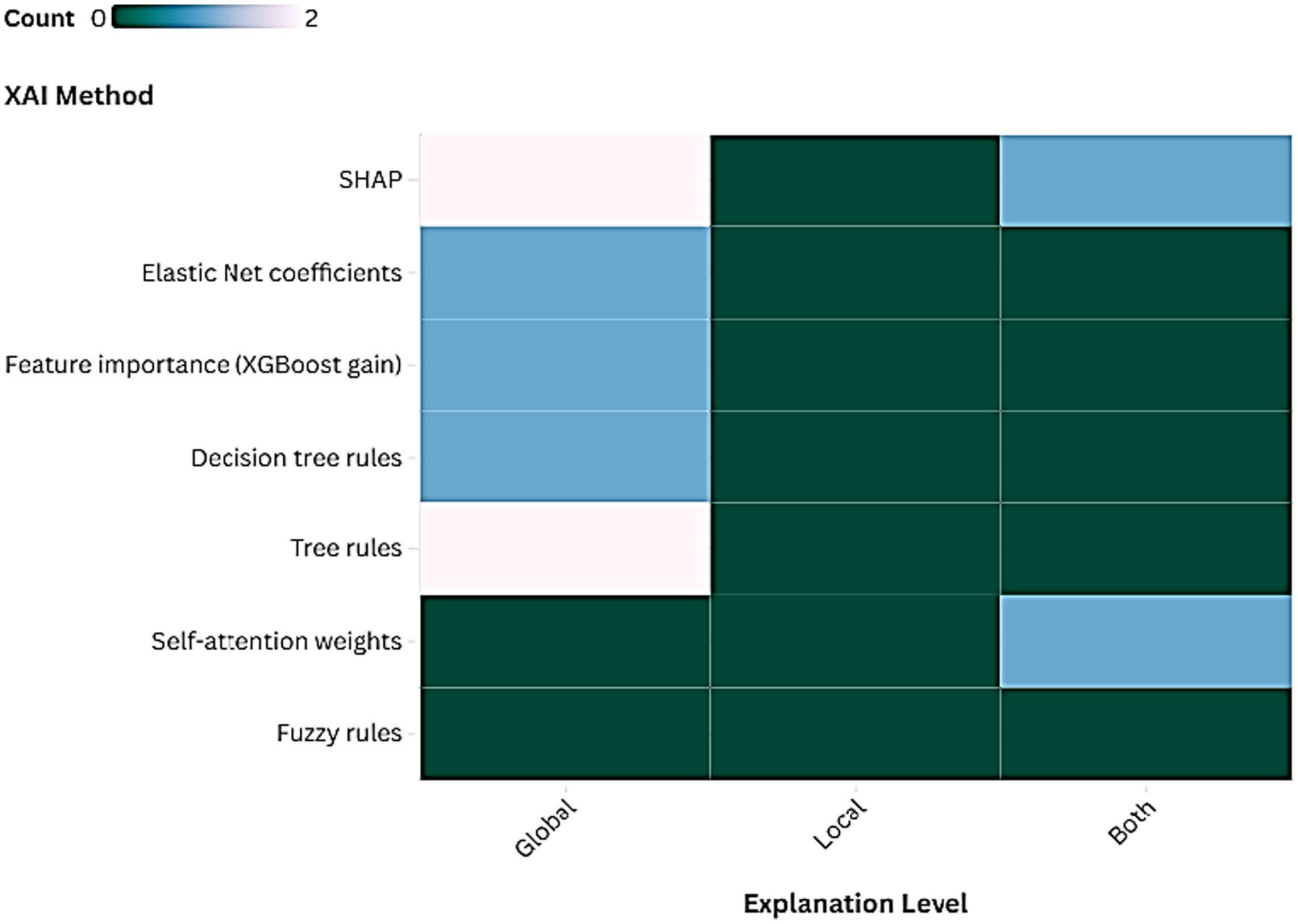
Heatmap of XAI methods by explanation level (global, local, both) across included studies, illustrating the predominance of global explainability approaches and limited use of combined global–local explanations.

Explainability output was mostly provided in the form of model-level summaries that were designed to provide a sense of what inputs tended to be important to predicting fall risks, and not to provide patient-specific reasonings. The heatmap (Figure 5) summarises the relationship between XAI methods and the level of explanation. The most common level of explanation was the global one, at which the interpretability was expressed in the form of rules, model coefficients, structures of risk stratification, or visual summary of importance, supporting the widespread clinical sense-making of the determinants of fall risk (Sato and Watanabe, 2025; Makino et al., 2021; Wang et al., 2024; Noh et al., 2021; Liang et al., 2024; Fong et al., 2023; Gillain et al., 2019; Schniepp et al., 2021). The meaning of the explanation used in such studies mostly corresponded to the discovery of salient gait/clinical predictors or explicit decision pathways (e.g., decision trees) that could be related to existing clinical constructs.

A smaller group of studies offered explanations that may be viewed as the association of risk to structured clinical categories. As an example, certain interpretable models gave decision rules or risk-group stratification results, which could be discussed directly as screening-relevant logic (Makino et al., 2021; Fong et al., 2023; Gillain et al., 2019), whereas regression-based methods offered coefficient-based interpretability to facilitate clinical interpretation of predictor-outcome relationships (Wang et al., 2024; Schniepp et al., 2021). These forms of explanation are more conducive to general clinical plausibility and communication of contributing factors, although they still are mainly suited to the level of model behaviour, as opposed to individual justification of decision.

Two studies gave both global and local explanation outputs. Mishra et al. used SHAP to generate global feature-importance explanations as well as local, patient-specific explanations showing which variables increased or decreased an individual’s fall risk prediction (Mishra et al., 2022). Similarly, El Marhraoui et al. employed a CNN-based self-attention architecture that generated interpretable temporal weight maps highlighting specific moments within IMU sensor recordings associated with elevated fall probability. These visual explanations enabled interpretation of “when” risky moments occurred during movement by identifying short time intervals with abrupt acceleration changes linked to balance loss and potential fall events (El Marhraoui et al., 2023). Table 2 sums up the different strategies of explainability by type of model.

**Table 2:** Relationship between model category and explainability strategy across included studies, showing how intrinsic and *post-hoc* XAI methods are applied at different explanation levels (global, local, or both).

Model	Category	XAI method	Level
Mohan et al. (2025)	Hybrid	Fuzzy rules	Not reported
Sato and Watanabe (2025)	Hybrid	SHAP	Global
Wang et al. (2024)	Interpretable	Elastic Net coefficients	Global
El Marhraoui et al. (2023)	Hybrid	Self-attention weights	Both
Schniepp et al. (2021)	Interpretable	Logistic regression coefficients/odds ratios	Global
Noh et al. (2021)	Black box	Feature importance (XGBoost gain)	Global
Makino et al. (2021)	Interpretable	Decision tree rules	Global
Gillain et al. (2019)	Interpretable	Tree rules	Global
Liang et al. (2024)	Hybrid	SHAP	Global
Fong et al. (2023)	Interpretable	Tree rules	Global
Mishra et al. (2022)	Hybrid	SHAP	Both

### 3.12 Explainability strategies by model type

**Figure 6:**
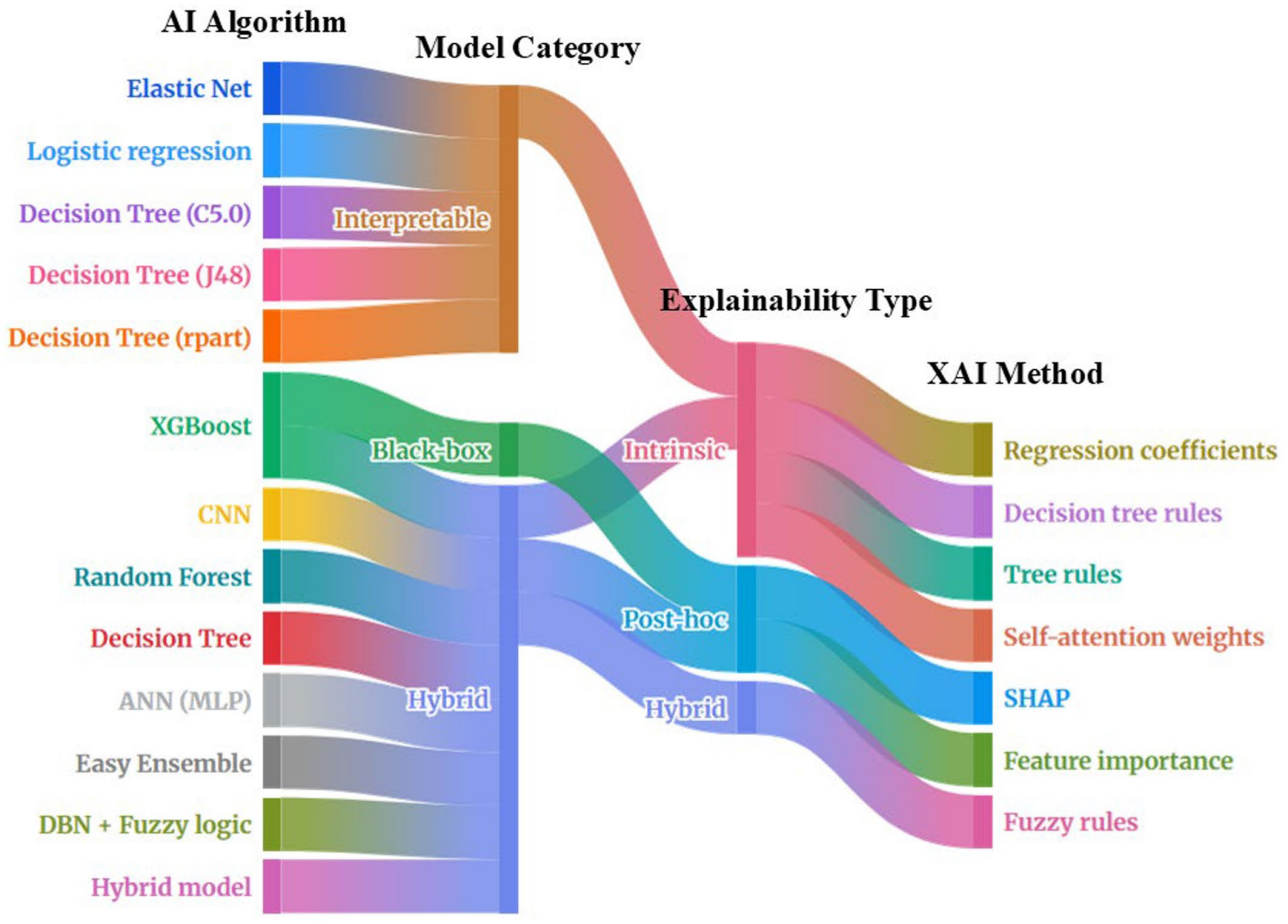
Alluvial diagram showing relationships between machine-learning algorithms, model interpretability categories, explainability approaches, and XAI techniques used in the included studies. Each flow represents one study–algorithm combination reported in the included studies. RF, Random Forest; XGBoost, Extreme Gradient Boosting; DT, Decision Tree; ANN, Artificial Neural Network; DBN, Deep Belief Network; CNN, Convolutional Neural Network; SHAP, Shapley Additive Explanations.

In all the studies included, machine-learning algorithms were mapped to various categories of interpretability and explainability strategies (Figure 6). The main frameworks upon which intrinsically interpretable models were built were decision trees and regression-style classifiers, which allowed extracting decision rules or model coefficients directly (Makino et al., 2021; Wang et al., 2024; Fong et al., 2023; Gillain et al., 2019; Schniepp et al., 2021). These strategies were based on inherent explainability by tree rules or coefficient exploration as opposed to external *post-hoc*. Conversely, black-box and hybrid models such as ensemble learners and neural networks were more often related to *post-hoc* explainability methods. SHAP-based feature attribution models were usually used to interpret random forest, gradient-boosting, and multilayer perceptron models (Sato and Watanabe, 2025; Liang et al., 2024), whereas gain-based feature importance measures were applied to XGBoost models (Noh et al., 2021).

In general, the patterns of flows suggest that intrinsically explainable algorithms made use of intrinsic explanation algorithms whereas complex ensemble and neural network models depended on *post-hoc* XAI techniques such as SHAP or feature importance.

### 3.13 Validation strategies

Validation was exclusively internal across all included studies; no study performed independent external validation on a dataset from a separate site or period. Hold-out splits (70/30 to 80/20) were used in five studies (Liang et al., 2024; Fong et al., 2023; El Marhraoui et al., 2023; Mishra et al., 2022; Mohan et al., 2025), while cross-validation was employed in three others, most commonly 10-fold (Makino et al., 2021; Gillain et al., 2019; Mishra et al., 2022), with one study using stratified 5-fold cross-validation with grid-search optimisation (Sato and Watanabe, 2025) and another using Monte Carlo cross-validation with 50 repetitions (Wang et al., 2024). One study incorporated prospective six-month follow-up data as part of model development (Schniepp et al., 2021), though this remained a form of internal rather than external validation.

### 3.14 Predictive performance

**Figure 7:**
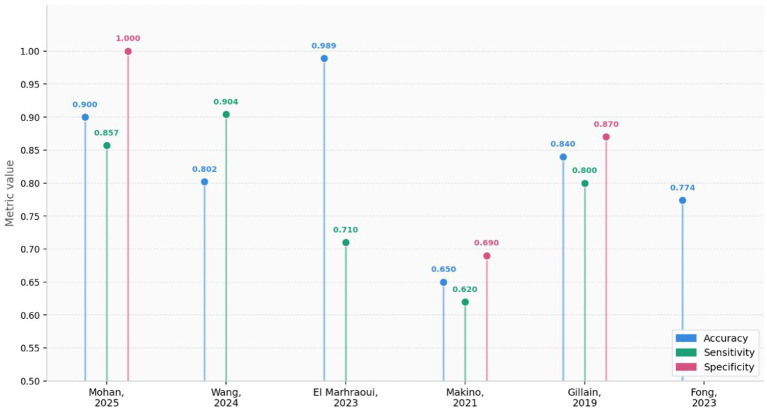
Summary of reported model performance across included studies, showing accuracy, sensitivity, and specificity for fall risk assessment tasks.

Predictive performance varied considerably across studies, tasks, and model types (Figure 7), with accuracy ranging from approximately 0.65 to 0.99. Interpretable models achieved moderate-to-high accuracy [0.65–0.92 (Makino et al., 2021; Gillain et al., 2019; Schniepp et al., 2021); 0.80 ± 0.16 for elastic net classification (Wang et al., 2024)]. Hybrid models generally outperformed these: a cooperative meta-model achieved 90% accuracy on three-class fall risk categorisation (Mohan et al., 2025), and attention-based CNN reached 98.9% for momentary balance-loss detection (El Marhraoui et al., 2023). The single black-box model achieved accuracy of 0.67–0.70 and AUC of 0.71–0.72 (Noh et al., 2021), while posturographic classifiers ranged from 0.76–0.90 (Liang et al., 2024) and a decision-tree model reported faller classification accuracy of 77.4% (Fong et al., 2023).

Sensitivity and specificity were inconsistently reported. Where available, sensitivity ranged from 0.43 to 0.96 (Sato and Watanabe, 2025; Noh et al., 2021) and specificity from 0.77 to 0.95 (Noh et al., 2021; Schniepp et al., 2021), with AUC values of approximately 0.70–0.90 (Makino et al., 2021; Noh et al., 2021; Liang et al., 2024).

### 3.15 Deployment and real-time readiness

The models that were evaluated in most of the studies were in offline context, there was no direct implementation in real time clinical/wearable systems. The processing location carried out was hardly ever reported, but when it was, the processing was performed using cloud-based or offline analytical environments and not on-device processing (Liang et al., 2024; Fong et al., 2023; Gillain et al., 2019; Mohan et al., 2025).

Performing in real-time was typically unreported or was specifically mentioned as being absent (Sato and Watanabe, 2025) and (El Marhraoui et al., 2023) examined recorded structured walking offline for short duration without inference in real-time (Mohan et al., 2025). Used the concept of multimodal data streams processing through clouds, although they did not mention real-time operation. Model evaluation was also done offline in studies based on retrospective clinical information or laboratory gait experiments (El Marhraoui et al., 2023; Mohan et al., 2025).

### 3.16 Risk of bias analysis

**Figure 8:**
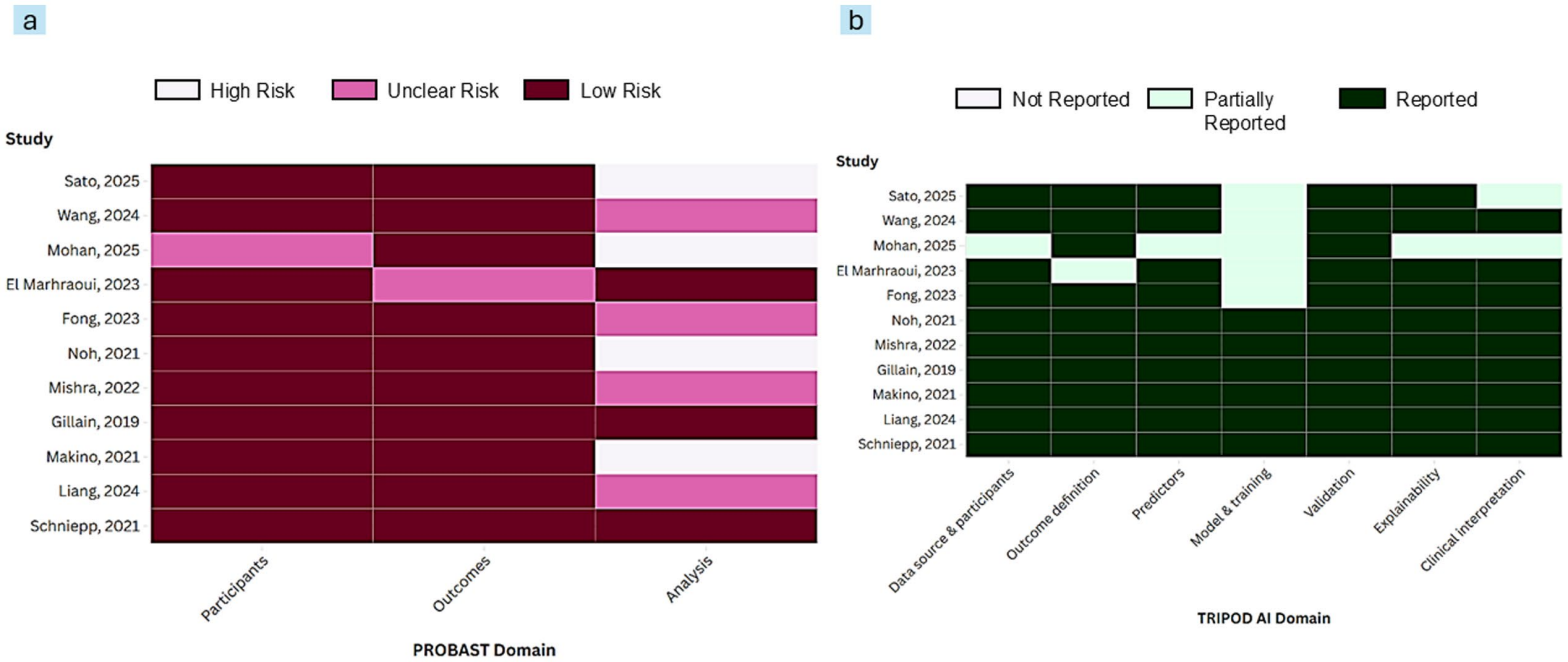
**(a)** Heatmap illustrating domain-level risk of bias across included studies, evaluated using PROBAST. Domains include participants, outcomes, and analysis. Risk of bias was encoded numerically with a three-colour encoding. **(b)** Heatmap summarising the TRIPOD AI domains (participants, predictors, outcome, and analysis) for each included study. Dark green indicates ‘reported’, light green indicates ‘partially reported’, and white indicates ‘not reported’.

With a modified PROBAST framework, based on the Domains of Participants, Outcomes and Analysis, most of the studies were rated as having a *low risk of bias* in terms of participant selection and definition of outcomes (see Figure 8a). Conversely, all the studies were rated as *high risk of bias* in the Analysis domain mainly because they used internal validation as a means of validation, had lack of external validation, and small sample sizes in comparison with model complexity.

### 3.17 Reporting quality

The quality of reporting in all the included studies was *low-moderate* to *moderate-good* (Figure 8b). Most of the studies sufficiently presented their data sources and the characteristics of the participants, outcomes, and predictor variables. The process of model specification and training was often partially reported especially in hybrid or multi-component frameworks (El Marhraoui et al., 2023; Mohan et al., 2025).

Every study had some type of internal validation, the most frequent method of which was cross-validation or hold-out splits, although no study had external validation. Explainability methods were in general well-reported, and most of the studies reported intrinsic explanations (e.g., coefficients or decision rules), or *post-hoc* (e.g., SHAP or feature importance), mostly at the global level.

Clinical interpretation was typically addressed, with the identified predictors related to the known factors of falls- risk, but this has not been adequately reported in other studies (Sato and Watanabe, 2025; Mohan et al., 2025). The completeness of reporting was supported on an ordinal scale (Figure 8b).

## 4 Discussion

### 4.1 Principal findings and contribution of this review

The synthesised evidence presented in this systematic review is on interpretable and explainable artificial intelligence (AI) models of wearable sensor-based falls-risk assessment among the elderly, alongside a clear focus on clinical interpretability and translational relevance. In the 11 included studies, a number of common patterns were identified: (i) wearable inertial sensing and laboratory-based gait measurements were the most common; (ii) there is a high heterogeneity in the definitions of fall risk and fall risk prediction tasks; (iii) intrinsically interpretable or hybrid modelling methods were widely used; and (iv) methodological immaturity on validation, explainability analysis, and field applications. To the best of our knowledge, no prior review has formally synthesised wearable-sensor-based fall risk assessment through the lens of interpretability and explainability. Although P&O practice represents a clinically important downstream application of this evidence base, it is important to note that none of the 11 included studies recruited or stratified P&O users, and no device-specific predictors were extracted; the P&O discussion in this review therefore constitutes an evidence-informed translational extrapolation rather than a direct empirical finding.

**Figure 9:**
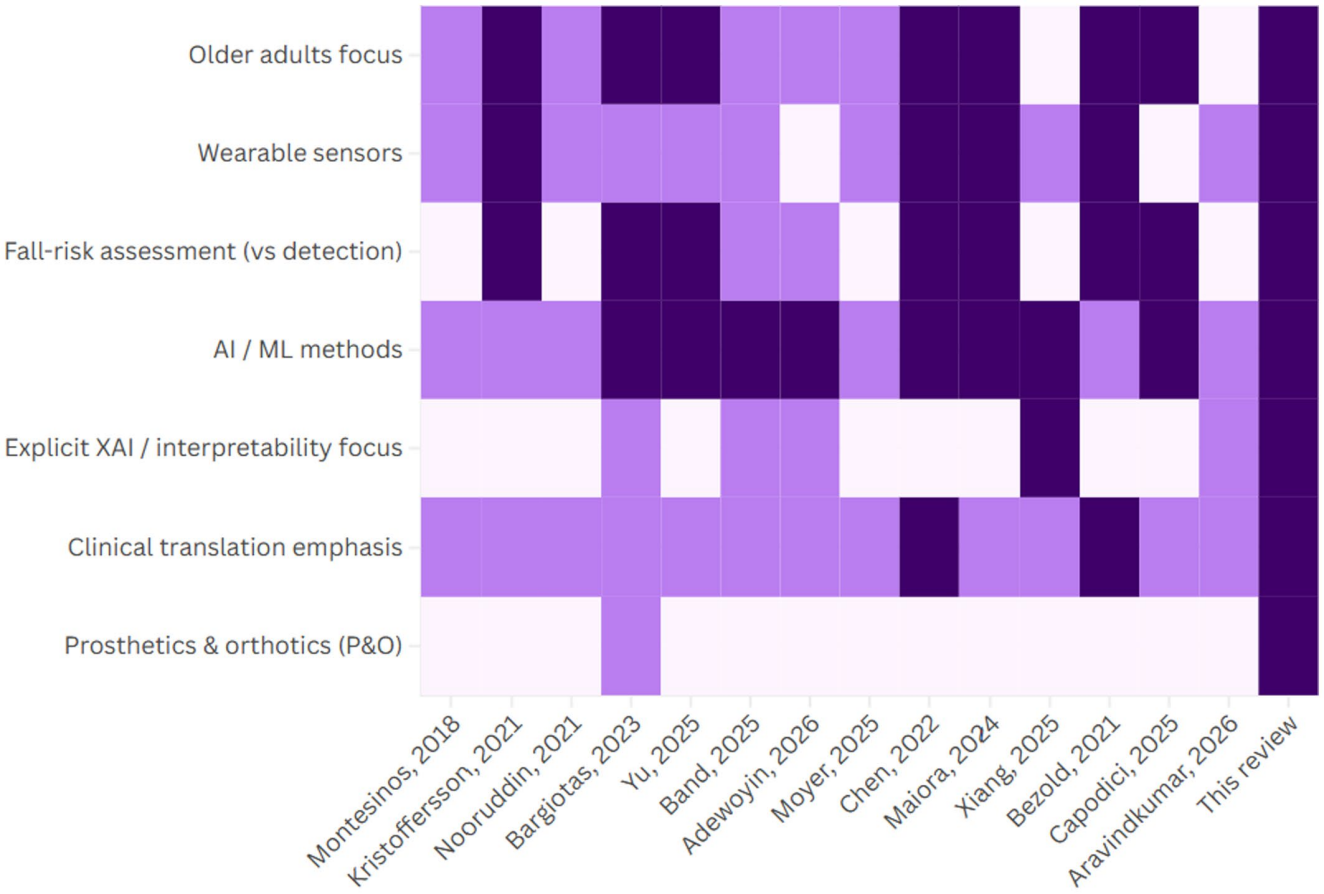
Heatmap comparing the thematic coverage of previous reviews and the present review across population, technology, methodological, explainability, and clinical focus domains. Values indicate absence (0), partial coverage (1), or extensive coverage (2).

Although the use of interpretable and explainable AI in wearable sensor-based fall risk assessments has been increasing in the older adult population, this area is emerging. There is currently no systematic review to synthesize the evidence at the intersection between wearable sensors, fall risk assessment (as opposed to detection), older adult populations, including those using prosthetic and orthotic devices, and in an interpretable or explainable manner. This gap is illustrated in Figure 9 which compares the scope of existing reviews and highlights the unique contribution of the present review.

The principal findings of this review, that inertial wearable sensors dominate data collection, that fall risk definitions remain highly heterogeneous, that intrinsically interpretable or hybrid models are preferred over black-box approaches, and that methodological immaturity persists in validation and explainability practice, are each corroborated and contextualised by the broader literature. The predominance of inertial measurement units is well-established: Chen et al. identified IMU-based sensing as the dominant modality across 25 wearable fall risk studies and demonstrated that model accuracy is systematically influenced by sensor location, type, and feature engineering (Chen et al., 2022). Maiora et al. showed that IMU signals captured during the Timed Up and Go test are sufficient to train deep learning fall risk models (Maiora et al., 2024). Capodici et al. also identified a lack of transparent reporting and standardisation as pervasive limitations even in sensor-free machine learning fall risk models (Capodici et al., 2025). Xiang et al.’s 2025 systematic review of XAI in gait analysis found that model-agnostic *post-hoc* techniques were more common than intrinsic interpretability approaches across 31 studies, and that clinical utility of explanations remained largely undemonstrated (Xiang et al., 2025), a finding echoed by a systematic review which observed that explanation evaluation across diagnostic AI studies is typically limited to descriptive summaries or isolated case illustrations rather than standardised assessment of faithfulness or stability (Aravindkumar et al., 2026). The preference for transparent over black-box architectures observed across the studies in the present review is also observed in computational-neuroscience literature on ageing. Mahamadou et al., for instance, used interpretable architectures combining generalised additive models with boosted trees to characterise nonlinear predictors of cognitive ageing while retaining the transparency required for clinical adoption (Mahamadou et al., 2025).

Interestingly, none of the 11 studies evaluated in this review explicitly recruited or stratified participants with a prosthetic or orthotic device and did not use predictors or moderators (e.g., alignment parameters, component stiffness, socket fit measures, or compensatory gait adaptations). This lack of evidence points to a significant evidence gap, namely, general older-adult populations can serve to inform the background of XAI to gait and balance monitoring, but the specific biomechanical asymmetries, altered loading patterns, and device-user interactions, which are inherent to P&O users, are still not directly addressed in interpretable ML/XAI frameworks with the help of wearable sensors. This gap needs to be addressed with specific, prosthesis/orthosis-focused research which will allow converting explainable models into clinically practical applications, which can be used by prosthetists, orthotists, and rehabilitation teams.

### 4.2 Machine-learning algorithms for wearable sensor-based fall risk assessment

In the studied literature, the fall risk assessment with wearable sensor data was widely carried out with classical machine-learning algorithms, such as logistic regression, elastic net, decision trees, random forests, and gradient-boosted ensembles. It is also important to distinguish these approaches from fall detection systems, which focus on real-time event identification rather than longitudinal risk estimation (Zhang et al., 2026). Deep learning models were relatively uncommon and, when existent, were often limited to hybrid designs with attention mechanisms and not end-to-end models. Early studies using inertial sensor–derived spatiotemporal gait parameters with deep learning models demonstrated strong predictive performance, highlighting the potential of such approaches when combined with domain-informed features (Tunca et al., 2020). Such a tendency is indicative of a methodological decision: to make the models transparent, resistant to small samples, consistent with clinically interpretable characteristics rather than being as predictive as possible.

This choice is quite justified in terms of machine-learning since wearable sensor data are not uniform in fall risk studies. The size of most datasets was small, heterogeneous in sampling frequency, and based on short-duration laboratory tasks instead of continuous free-living monitoring. In this case, simplified models with explicit features representations are less subjected to overfitting, and the attribution of risk to biomechanical variables becomes less ambiguous.

Across the included studies, interpretable models, logistic regression, elastic net, and decision trees, achieved accuracy ranging from 0.65 to 0.92 (Makino et al., 2021; Wang et al., 2024; Fong et al., 2023; Schniepp et al., 2021), with AUC values of 0.70–0.92 where reported. Hybrid and black-box designs, XGBoost, ensemble balancing classifiers, and CNN with attention, produced accuracy of 0.67–0.99 (Noh et al., 2021; Liang et al., 2024; El Marhraoui et al., 2023; Mohan et al., 2025). The upper bound of the hybrid range, however, requires qualification: the 98.9% accuracy reported by El Marhraoui et al. (El Marhraoui et al., 2023) derives from a binary balance-loss detection task on short laboratory sequences in a single clinical cohort, a considerably more constrained problem than the prospective fall risk classification attempted by the interpretable studies; when hybrid models are compared against interpretable models on structurally equivalent tasks, community-based prospective classification or cross-sectional risk stratification, the performance differential narrows substantially, with overlapping ranges of 0.67–0.77 for hybrid designs (Noh et al., 2021; Liang et al., 2024) against 0.65–0.84 for interpretable ones. The direct head-to-head comparisons conducted within individual studies reinforce this convergence: Wang et al. (Wang et al., 2024) demonstrated that ensemble elastic net matched or outperformed SVM, KNN, and naïve Bayes on accuracy and sensitivity while retaining full coefficient-level interpretability; Fong et al. (Fong et al., 2023) found that a decision tree achieved 77–89% accuracy for faller classification where random forest variants produced 55–84% depending on sampling strategy; and Makino et al. (Makino et al., 2021) showed that a simplified 14-node decision tree with six clinical predictors outperformed logistic regression on both accuracy and AUC in a cohort of over 2,500 community-dwelling older adults. Taken together, these results provide a methodological argument for preferring interpretable designs in this domain that goes beyond pragmatic considerations of sample size and overfitting resistance. Where discriminative performance is statistically equivalent, interpretability is not a trade-off against predictive validity but an additional property obtained at no measurable cost, and in clinical contexts where a model’s output must be actionable by a practitioner, auditable under governance frameworks, and meaningful to the patient, it is the more consequential property of the two. The persistent assumption that black box complexity is a prerequisite for adequate discrimination is not supported by the evidence in this review, and the design choices of most included studies, favouring transparent architectures without sacrificing performance, reflect a considered methodological position that this field is well-placed to consolidate.

Nevertheless, one limitation that was always present was the limited modelling of temporal structure. Despite the inherent time-series nature of wearable sensors, most of the studies did not make use of time dynamics per se, instead utilized summary statistics (e.g., mean gait speed, variability measures, postural sway indices). Newer methods that model the full gait cycle over time show that paying attention to how movement patterns change moment to moment can improve both prediction and clinical usefulness (Bian et al., 2025). Yet only one of the reviewed studies used attention-based temporal modelling, so there is a clear missed opportunity here, particularly for capturing the shifting instability and fatigue that build up as someone walks. This matters especially for people using a prosthesis or orthosis. Other areas of elderly care are already moving in this direction: researchers are building attention-based models that combine several data sources to track how a patient’s physiological signals change over time (Xinli et al., 2025). The fall-risk field is relevantly novice in this kind of approach.

### 4.3 Explainability and interpretability: existing practices and limitations

Two general strategies were used to achieve explainability: intrinsically interpretable models (e.g., regression coefficients, decision tree rules), and *post-hoc* explanations methods, most commonly SHAP and feature importance scores. The interpretation of results was mainly intrinsic, while *post-hoc* techniques were fewer and were usually applied on a global scale.

From an explainable machine-learning standpoint, this reflects a conservative but sensible approach. Intrinsic models offer consistent, readily explained explanations, which are consistent with well-known biomechanical rationale, including the significance of gait speed, stride variability, or balance-related characteristics. Emerging hybrid interpretable architectures further indicate that complex models can generate clinically meaningful rules through embedded reasoning mechanisms, challenging the traditional trade-off between interpretability and performance (Zhang et al., 2025). These descriptions are also useful, especially in clinical practices where they require trust, transparency, and auditing.

However, the review reveals certain significant shortcomings. To begin with, explainability was not often addressed as a model output that should be assessed but merely provided. Not many studies evaluated the stability of explanations, sensitivity to perturbations in inputs, or consistency across validation folds. Secondly, the explanations were overwhelming at the population-level, which gives the insights on the average feature relevance but not on an individual risk attribution. To perform fall risk assessment, and particularly prosthetics and orthotics applications, there must be instance-level explanations to facilitate individualised clinical decision-making.

Of the 11 included studies, two (El Marhraoui et al., 2023; Mishra et al., 2022) provided both global and local explanations, combining population-level mean SHAP rankings with instance-level decompositions stratified by gait phenotype. The remaining nine studies reported exclusively global outputs: aggregate feature-importance rankings, population-level decision-tree rules, or mean absolute SHAP values summarised across the full cohort. This constitutes a critical methodological gap for any clinical translation, but it is particularly consequential in P&O practice, where the unit of clinical decision-making is invariably the individual patient rather than the population. A global feature-importance ranking communicates that stride variability is, on average, a stronger fall risk driver than cadence across a study cohort; it cannot communicate whether, for this patient on this occasion, the dominant risk contribution derives from prosthetic inertia amplifying swing-phase timing, from socket discomfort altering weight-acceptance strategy, or from a neurological deficit that predates the device prescription. These are mechanistically distinct causes that warrant entirely different clinical responses, component adjustment, socket modification, or onward neurological referral respectively, and no global explanation, however detailed, can resolve between them at the individual level. Instance-level methods such as local SHAP, LIME, or temporal attention decomposition generate exactly this resolution: a ranked attribution of risk to features for a specific patient at a specific measurement occasion, which a prosthetist or orthotist can interrogate in the same way they interrogate a gait analysis report. The absence of such outputs from nine of 11 reviewed studies is not merely a reporting limitation; it reflects a design choice, prioritising population-level model evaluation over individual-level clinical utility, that would need to be deliberately reversed in any P&O-facing implementation. Addressing this gap does not necessarily require more complex models: the elastic net (Wang et al., 2024), decision tree (Gillain et al., 2019), and SHAP-augmented ensemble (Liang et al., 2024) architectures already present in this review are all capable of generating local explanations, and their failure to do so represents an unrealised translational opportunity rather than a technical barrier.

Moreover, explainability was not always a characteristic of sensor modality constraints. Wearable sensor features are inherently dependent on placement, orientation, and activity context. However, not many studies investigated whether the explanations could be meaningful in different sensor configurations or whether some modalities biased feature importance. This disconnect constrains the clinical viability of explainable AI outputs that may be required when projecting results across devices or settings.

### 4.4 Applicability of wearable sensor modalities within ML pipelines

The most common source of data was wearable inertial sensors, especially IMU, due to their cost-effectiveness, feasibility, and proven usefulness in gait analysis. Features that were generated by sensors are generally presented to machine-learning models as pre-engineered inputs, which enhances the interpretability of the modelling pipeline.

In terms of ML systems, this sensor-feature-model architecture is the best: domain knowledge can influence the process of feature extraction, and the learned relations can be physically plausible. Nonetheless, it further limits model expressiveness, and might mask delicate time trend related to imminent instability or context-specific fall risk.

Notably, most of the studies used either single-session or laboratory sensor records and thus restricted the variety of movement patterns during training. Evidence from longitudinal ambient monitoring systems suggests that continuous, context-aware data collection, rather than sensor modality alone, may be a key determinant of predictive performance in fall risk modelling (Adeli et al., 2023). Due to this, machine-learning models can inadvertently acquire task-specific, and not behaviour-general representations. This constraint has a direct effect on explainability, since explainability based on restricted tasks might not transfer to the real-world situations in which falls do indeed happen.

### 4.5 Clinical implications for prosthetics and orthotics

#### 4.5.1 Scope and necessary caveat

None of the 11 included studies recruited prosthesis or orthosis users or incorporated device-specific covariates. The discussion that follows is an evidence-informed extrapolation and must be read as a platform for future research rather than a finding of this review. Lower-limb prosthesis and orthosis users present biomechanical profiles that differ structurally from the populations on which the reviewed models were trained: obligatory inter-limb asymmetries, altered loading on both the residual and intact limb, and compensatory postural strategies interact with sensor-derived features in ways that able-bodied training data cannot represent. A model deployed without modification in a P&O clinic would encounter a distribution shift sufficient to invalidate both its calibration and its XAI-derived feature attributions. The value of engaging with this evidence base lies in extracting the mechanistic logic that XAI makes explicit and using it to specify what a P&O-specific implementation would require. The full predictor-to-mechanism mapping is provided in Supplementary Table S4.

#### 4.5.2 XAI-identified predictors and P&O mechanisms

##### 4.5.2.1 Gait speed, stride length, and the device-configuration confound

Gait speed was the most consistently identified predictor across the reviewed XAI outputs (Sato and Watanabe, 2025; Noh et al., 2021; Fong et al., 2023; Gillain et al., 2019). In able-bodied older adults it reflects a composite of neuromuscular and cardiorespiratory decline. In prosthesis users it is additionally modulated by sagittal alignment errors, socket pistoning, and foot-ankle stiffness, all remediable through fitting. An XAI model flagging slow gait speed as the primary risk driver in a prosthesis user may therefore be generating a correct statistical association but a misleading clinical inference the speed deficit may be correctable rather than intrinsic. Stride length carries the same confound, since elastic net or decision-tree thresholds derived from able-bodied data will penalise a prosthetic foot energy-return deficit as though it were equivalent to sarcopenic weakness. Instance-level SHAP applied to a model that includes socket comfort ratings, foot-component category, and alignment offset as explicit inputs would allow attribution to be decomposed between patient-intrinsic and device-attributable components, a capability absent from all 11 included studies.

##### 4.5.2.2 Stride variability, stance asymmetry, and the baseline-elevation problem

Stride-time variability and double-support duration were significant predictors (Wang et al., 2024; Noh et al., 2021; Liang et al., 2024; Gillain et al., 2019). In prosthetic gait, variability is structurally elevated above able-bodied norms due to prosthetic inertia, asymmetric propulsion, and degraded sensory feedback from the residual limb. Thresholds derived from able-bodied training data will therefore produce a systematic specificity deficit, classifying a disproportionate fraction of prosthesis users as high-risk on variability grounds irrespective of their actual fall history. Asymmetry-adjusted reference ranges, derived from a prosthesis-user cohort and validated through XAI residual analysis, comparing feature attributions from able-bodied-trained and prosthesis-trained models on the same test set, would address this problem directly. Prolonged double-support time should similarly be treated as an adaptive compensation with population-specific reference values rather than a shared pathological threshold.

##### 4.5.2.3 Balance features, functional composites, and orthotic device effects

BBS scores, TUG performance, postural sway, and trunk acceleration indices (Wang et al., 2024; Liang et al., 2024; Fong et al., 2023; El Marhraoui et al., 2023; Gillain et al., 2019) are directly modified by orthotic ankle stiffness, prosthetic foot roll-over shape, and socket fit quality, parameters that change between fitting appointments and vary across component prescriptions. The TUG sub-task segmentation of Wang et al. (2024) is particularly instructive: the turning phase is disproportionately sensitive to prosthetic rotational resistance, while sit-to-stand reflects socket brim fit and hip extensor loading. Phase-specific SHAP attribution could localise a functional deficit to a specific gait event and connect it to the most likely device parameter. The temporal attention heatmaps of El Marhraoui et al. (2023) extend this logic to orthotics, where heel-strike transients amplified by orthotic heel stiffness and toe-off instability from insufficient plantarflexion assistance are identifiable as discrete high-risk windows within a movement trial.

#### 4.5.3 Longitudinal XAI tracking as a device-fitting outcome metric

A third application, absent from the current literature, is the use of longitudinal XAI feature-importance trajectories as quantitative outcome metrics for fitting interventions. Conventional outcome measurement in P&O, repeated TUG, PEQ, captures net change but cannot attribute change to specific biomechanical mechanisms. If an interpretable model is applied at successive fitting appointments and SHAP weights are tracked over time, the trajectory of feature importance becomes an outcome variable. Under successful fitting, the expected shift is from balance-related features, postural sway, double-support time, BBS sub score deficits reflecting compensatory instability management, toward gait-timing and spatial features reflecting confident, propulsive locomotion. Conversely, a trajectory in which balance features increase in weight despite stable aggregate scores may signal incipient device degradation, socket volume loss, foot-component fatigue, orthotic material creep, before it reaches clinical detection threshold. The interpretable and hybrid models reviewed here (Sato and Watanabe, 2025; Wang et al., 2024; Liang et al., 2024; Gillain et al., 2019) all generate the feature-level outputs required for longitudinal tracking, provided they are applied to repeated individual-level measurements rather than once to a population cross-section.

#### 4.5.4 Research priorities

Realising these applications requires four conditions:

(i) Training on datasets that include device-state covariates, alignment parameters, component type, socket fit ratings, orthotic stiffness, alongside gait-sensor features, so that XAI outputs can partition risk attribution between modifiable device factors and patient-intrinsic characteristics.
(ii) Prioritising instance-level explanation methods (local SHAP, LIME, temporal attention) over global feature-importance rankings, and co-designing output interfaces with prosthetists and orthotists to ensure attributions are legible within clinical alignment reasoning workflows.
(iii) Establishing longitudinal XAI tracking protocols in which feature-importance trajectories are recorded as a primary or secondary outcome measure alongside conventional functional scores at each fitting appointment.
(iv) Validating asymmetry-adjusted variability thresholds in prosthesis-user cohorts using XAI residual analysis to quantify the magnitude of correction required relative to able-bodied reference models.

In addition to predictive validity and interpretability, successful translation will depend on the feasibility of real-time or near-real-time deployment on wearable or edge-computing platforms, where latency, computational efficiency, and continuous monitoring capability become critical determinants of clinical usability.

### 4.6 Review limitations

This review was not prospectively pre-registered in PROSPERO or any other systematic-review registry. Although the review methodology was developed *a priori* and conducted in accordance with PRISMA guidelines, the absence of protocol pre-registration may increase the risk of unintentional methodological bias and reduces transparency regarding potential *post-hoc* methodological refinements.

A notable inconsistency exists between the stated eligibility criteria and two of the included studies. The original eligibility criterion specified that studies must have “used wearable or hybrid sensor devices”; however, two studies (Makino et al., 2021; Fong et al., 2023) relied exclusively on structured clinical variables and did not involve wearable sensor devices. Rather than excluding these studies, which applied interpretable AI/ML models to clinically meaningful fall risk assessment tasks in older adults and satisfied all other eligibility criteria, the criterion was revised *post-hoc* to encompass wearable sensors, hybrid sensor systems, and structured clinical assessment instruments as permissible data sources. This revision broadens the review’s scope slightly beyond its original wearable-sensor focus. Readers should note that findings from these two studies pertain to clinically administered assessments rather than sensor-derived signals, and their inclusion should be considered when interpreting the generalisability of conclusions to purely wearable-based settings.

### 4.7 Future methodological directions

In future studies, machine-learning pipelines, which (i) combine wearable sensors data with device-specific variables (ii) leverage temporal modelling and retain interpretability, (iii) can provide sound instance-level explanations and (iv) can assess the utility of explanations in clinical practice, should be given a priority. The use of hybrid methods that combine explainable time-based models with wearable sensing is a potential field of interest, especially to measure fall risk dynamics when using a prosthesis and orthosis in the real world.

## 5 Conclusion

This systematic review synthesises evidence from 11 studies on interpretable and explainable AI for wearable sensor–based fall risk assessment in older adults and evaluates its translational relevance for prosthetics and orthotics practice. Three substantive conclusions emerge.

Interpretability is a viable and methodologically defensible design choice in this domain, not a concession. In direct within-study comparisons, interpretable models frequently achieved performance comparable to hybrid or black-box architectures on structurally equivalent tasks, and in several direct within-study comparisons matched or outperformed more complex alternatives. Where discriminative performance is statistically equivalent, interpretability is not a trade-off against predictive validity, but an additional property obtained at no measurable cost, and in clinical contexts where model outputs must be actionable, auditable, and meaningful to the patient, it is the more consequential property of the two.

The field has not yet achieved the methodological maturity required for clinical deployment. Every included study relied exclusively on internal validation, none performed external validation or real-time deployment, and nine of 11 studies provided only global explainability outputs. Global feature-importance summaries support model-level interpretation but cannot generate the patient-specific attributions required for individualised clinical decision-making. Progress toward deployment requires prospective studies with external validation cohorts, standardised outcome definitions, and systematic adoption of local explanation methods evaluated as primary model outputs rather than incidental by-products.

The translational pathway to P&O practice is conceptually well-founded but evidentially unestablished. None of the reviewed studies recruited prosthesis or orthosis users or incorporated device-specific predictors. Extending this evidence base to that population requires device-specific training data, asymmetry-adjusted feature thresholds, instance-level explanations, and clinician-facing output interfaces. Without these, explainable AI in this domain risks remaining a methodological demonstration rather than a functional decision-support tool, and the gap between those two outcomes defines the research agenda that must now be addressed.

## Data Availability

The original contributions presented in the study are included in the article/Supplementary material, further inquiries can be directed to the corresponding author.
