# Supplementary material for: Interpretable and explainable artificial intelligence for wearable sensor-based fall risk assessment in older adults: a systematic review with considerations for prosthetics and orthotics
Source: Front Comput Neurosci. 2026 Jul 14;20:1860978. doi: 10.3389/fncom.2026.1860978 (PMC13408035; doi:10.3389/fncom.2026.1860978)
Supplement: Supplementary file 1 [file Table_1.docx]

# **Supplementary Table S1. Full search strings used across databases**

| **Database** | **Search String** |
| --- | --- |
| **PubMed** | ("Aged"[Mesh] OR elderly OR "older adults" OR seniors OR geriatric OR ageing OR aging) AND ("Accidental Falls"[Mesh] OR "fall risk" OR falls OR falling OR "fall-risk assessment" OR "fall prediction") AND ("Artificial Intelligence"[Mesh] OR "Machine Learning"[Mesh] OR "machine learning" OR "deep learning" OR "artificial intelligence" OR "predictive model*" OR "classification model*") AND (interpretable OR explainable OR explainability OR "explainable artificial intelligence" OR "explainable machine learning" OR XAI OR "transparent model*" OR "interpretable model*") AND (wearable OR "wearable sensors" OR accelerometer OR gyroscope OR "inertial sensor*" OR IMU OR "inertial measurement unit" OR "pressure sensor*" OR "smart insole*" OR "wearable device*") |
| **Scopus** | TITLE-ABS-KEY ((elderly OR "older adults" OR seniors OR geriatric OR ageing OR aging) AND ("fall risk" OR falls OR falling OR "fall-risk assessment" OR "fall prediction") AND ("machine learning" OR "artificial intelligence" OR "deep learning" OR "predictive model*" OR "classification model*") AND (interpretable OR explainable OR explainability OR "explainable artificial intelligence" OR "explainable machine learning" OR XAI OR "transparent model*" OR "interpretable model*") AND (wearable OR "wearable sensors" OR accelerometer OR gyroscope OR "inertial sensor*" OR IMU OR "inertial measurement unit" OR "pressure sensor*" OR "smart insole*" OR "wearable device*")) |
| **Web of Science Core Collection** | TS=((elderly OR "older adults" OR seniors OR geriatric OR ageing OR aging) AND ("fall risk" OR falls OR falling OR "fall-risk assessment" OR "fall prediction") AND ("machine learning" OR "artificial intelligence" OR "deep learning" OR "predictive model*" OR "classification model*") AND (interpretable OR explainable OR explainability OR "explainable artificial intelligence" OR "explainable machine learning" OR XAI OR "transparent model*" OR "interpretable model*") AND (wearable OR "wearable sensors" OR accelerometer OR gyroscope OR "inertial sensor*" OR IMU OR "inertial measurement unit" OR "pressure sensor*" OR "smart insole*" OR "wearable device*")) |
| **IEEE Xplore** | (("All Metadata":"elderly" OR "All Metadata":"older adults" OR "All Metadata":"seniors" OR "All Metadata":"geriatric" OR "All Metadata":"ageing" OR "All Metadata":"aging") AND ("All Metadata":"fall risk" OR "All Metadata":"falls" OR "All Metadata":"falling" OR "All Metadata":"fall-risk assessment" OR "All Metadata":"fall prediction") AND ("All Metadata":"machine learning" OR "All Metadata":"artificial intelligence" OR "All Metadata":"deep learning" OR "All Metadata":"predictive model" OR "All Metadata":"classification model") AND ("All Metadata":"interpretable" OR "All Metadata":"explainable" OR "All Metadata":"explainability" OR "All Metadata":"explainable artificial intelligence" OR "All Metadata":"explainable machine learning" OR "All Metadata":"XAI" OR "All Metadata":"transparent model" OR "All Metadata":"interpretable model") AND ("All Metadata":"wearable" OR "All Metadata":"wearable sensors" OR "All Metadata":"accelerometer" OR "All Metadata":"gyroscope" OR "All Metadata":"inertial sensor" OR "All Metadata":"IMU" OR "All Metadata":"inertial measurement unit" OR "All Metadata":"pressure sensor" OR "All Metadata":"smart insole" OR "All Metadata":"wearable device")) |
