# Supplementary material for: Interpretable and explainable artificial intelligence for wearable sensor-based fall risk assessment in older adults: a systematic review with considerations for prosthetics and orthotics
Source: Front Comput Neurosci. 2026 Jul 14;20:1860978. doi: 10.3389/fncom.2026.1860978 (PMC13408035; doi:10.3389/fncom.2026.1860978)
Supplement: Supplementary file 2 [file Table_2.docx]

Supplementary Table S2: Completed PRISMA 2020 checklist for the manuscript.

| **Section/Topic** | **Item No.** | **Checklist Item** | **Reported Location in Manuscript** |
| --- | --- | --- | --- |
| Title | 1 | Identify the report as a systematic review. | Title page |
| Abstract | 2 | Structured summary including objectives, methods, results, and conclusions. | Abstract |
| Rationale | 3 | Describe the rationale for the review. | Introduction |
| Objectives | 4 | Provide explicit statement of objectives/questions. | End of Introduction |
| Eligibility criteria | 5 | Specify inclusion and exclusion criteria. | Section 2.2 |
| Information sources | 6 | Specify databases and search dates. | Section 2.1 |
| Search strategy | 7 | Present full search strategies. | Supplementary Table S1 |
| Selection process | 8 | Specify screening methods and reviewers. | Section 2.1 |
| Data collection process | 9 | Specify extraction methods and reviewers. | Sections 2.1 and 2.4 |
| Data items | 10 | List and define extracted variables. | Section 2.4 |
| Study risk of bias assessment | 11 | Specify methods used to assess risk of bias. | Section 2.7 |
| Effect measures | 12 | Specify effect measures used. | Not applicable; qualitative synthesis |
| Synthesis methods | 13 | Describe synthesis methods. | Section 2.6 |
| Reporting bias assessment | 14 | Describe methods to assess reporting bias. | Not reported |
| Certainty assessment | 15 | Describe certainty assessment methods. | Section 2.8 |
| Study selection | 16 | Describe search and selection results. | Section 3.1 |
| Study characteristics | 17 | Cite and summarize included studies. | Sections 3.2 |
| Risk of bias in studies | 18 | Present risk of bias results. | Section 3.16 |
| Results of individual studies | 19 | Present summary data/results. | Sections 3.3–3.11 |
| Results of syntheses | 20 | Present synthesis results. | Sections 3.3–3.11 |
| Reporting biases | 21 | Present assessment of reporting bias. | Not reported |
| Certainty of evidence | 22 | Present certainty assessment. | Section 3.17 |
| Discussion | 23 | Interpret findings and limitations. | Section 4 |
| Limitations | 23c | Discuss limitations of evidence and review process. | Section 4 |
| Registration and protocol | 24 | Provide registration/protocol information. | Not reported (mentioned as a limitation) |
| Support | 25 | Describe funding/support. | Funding statement |
| Competing interests | 26 | Declare competing interests. | Conflict of interest statement |
| Availability of data/materials | 27 | Report availability of data and materials. | Supplementary tables S1 to S4 |
