# Supplementary material for: Interpretable and explainable artificial intelligence for wearable sensor-based fall risk assessment in older adults: a systematic review with considerations for prosthetics and orthotics
Source: Front Comput Neurosci. 2026 Jul 14;20:1860978. doi: 10.3389/fncom.2026.1860978 (PMC13408035; doi:10.3389/fncom.2026.1860978)
Supplement: Supplementary file 4 [file Table_4.docx]

**Supplementary Table S4: XAI-identified fall-risk predictors mapped to P&O mechanisms, clinical interpretive consequences, and proposed XAI applications. Studies cited by first author and year correspond to the included review studies.**

| **XAI-identified predictor** | **Domain** | **Source studies** | **P&O-relevant mechanism** | **Clinical consequence for P&O interpretation** | **XAI application in P&O practice** |
| --- | --- | --- | --- | --- | --- |
| Gait speed / walking speed | Temporal gait | Noh 2021; Sato 2025; Gillain 2019; Fong 2023 | Directly modulated by prosthetic alignment, socket pistoning, knee-moment-arm placement (transtibial), and foot-ankle stiffness. Orthotic stiffness gradients alter cadence-speed coupling. | Slow gait speed flagged as high-risk may reflect suboptimal device configuration rather than intrinsic decline. XAI feature weight for speed should be interpreted alongside device-parameter data. | Instance-level SHAP can distinguish speed reduction driven by socket discomfort (acute, modifiable) from neurological decline (chronic, less modifiable) if device covariates are included. |
| Stride length | Spatial gait | Noh 2021; Gillain 2019; Sato 2025 | Shortened by prosthetic foot energy-return deficit, residual-limb pain, and sagittal alignment errors that limit terminal stance push-off. | Elastic net / decision-tree rules penalising short stride may conflate energy-return limitations with neuromuscular weakness unless foot-component type is a covariate. | Longitudinal SHAP tracking: a shift from stride-length dominance toward balance-feature dominance post-alignment adjustment signals successful mechanical correction. |
| Stride-time variability / gait regularity | Temporal gait | Noh 2021; Gillain 2019; Liang 2024 | Prosthetic inertia (heavier distal components), asymmetric propulsion, and inter-limb loading asymmetry structurally amplify temporal variability above able-bodied norms. | Models trained on able-bodied older adults will systematically over-predict fall risk in prosthesis users due to baseline variability elevation; specificity will be low. | Asymmetry-adjusted variability thresholds derivable from XAI residuals; longitudinal reduction in variability weight post-fitting is a candidate device-fitting outcome metric. |
| Stance phase duration / double-support time | Spatial gait | Noh 2021; Wang 2024 | Prolonged double support is a hallmark of prosthetic gait adopted to compensate for reduced push-off and balance uncertainty on the prosthetic side. | Elastic net coefficients for double-support time will be confounded in P&O users; elevated double support is adaptive, not solely pathological. | Tracking double-support asymmetry index via XAI over a fitting episode quantifies whether alignment changes reduce compensatory reliance on the intact limb. |
| Postural sway / COP-based balance features | Balance / posturography | Liang 2024; El Marhraoui 2023; Mohan 2025 | Orthotic ankle stiffness directly constrains COP excursion range; prosthetic foot roll-over shape determines mediolateral sway. Socket fit affects proximal torque transmission and trunk sway. | SHAP values for sway features will be device-setting-dependent; the same patient may shift risk category with a stiffness adjustment. Static sway norms are not transferable from able-bodied populations. | Serial SHAP decomposition of sway features before/after orthotic adjustment provides a quantitative, explainable outcome measure beyond aggregate balance scores. |
| Trunk / ankle acceleration & jerk | Balance / posturography | El Marhraoui 2023; Wang 2024; Schniepp 2021 | Trunk acceleration is amplified by socket instability and energy-storing foot recoil; ankle jerk peaks are directly modulated by orthotic damping settings and prosthetic heel stiffness. | Self-attention temporal maps (El Marhraoui) highlighting high-jerk moments are interpretable as device-response events (heel strike transients) in prosthesis users. | Temporal attention heatmaps can localise specific gait events (heel strike, toe-off) where device-related instability peaks, actionable for component selection or alignment modification. |
| BBS-derived balance / functional balance score | Clinical / functional | Wang 2024; Fong 2023; Gillain 2019 | BBS subscores for standing on one leg and stepping are directly modified by prosthetic foot energy return and orthotic ankle stiffness. Socket comfort affects confidence and thus task performance. | BBS threshold rules (e.g. ≤50 = high risk) derived from able-bodied populations may misclassify prosthesis users whose BBS reflects device limitation rather than neuromuscular deficit. | Longitudinal decision-tree re-splitting on BBS subscores after component change identifies which balance tasks respond to device modification, a functional fitting outcome metric. |
| TUG score and sub-phases | Clinical / functional | Wang 2024; Fong 2023; Liang 2024 | TUG turning phase is disproportionately affected by prosthetic rotational resistance and knee-unit stability; sit-to-stand phase by socket brim fit and hip extensor loading. | Elastic net coefficients for TUG derived from able-bodied data cannot be directly applied; TUG sub-task segmentation (Wang 2024) provides the phase resolution needed to locate device-specific deficits. | Phase-specific TUG SHAP values tracked pre/post alignment: if turning-phase weight decreases after rotational alignment correction, XAI provides phase-resolved evidence of fitting benefit. |
| Fall history | Clinical / functional | Schniepp 2021; Fong 2023; Sato 2025; Makino 2021 | In P&O users, fall history encodes both intrinsic risk and historical device failures (socket loosening, component wear, ill-fitting orthoses). These are mechanistically distinct from neurological falls. | Regression models treating fall history as a homogeneous predictor will overestimate non-modifiable risk in P&O users whose past falls were device attributable. | Structured fall-event annotation (device-related vs. environmental vs. neurological) as an input covariate allows XAI to decompose fall-history weight into modifiable and non-modifiable components. |
| Fear of falling (FES-I) | Clinical / functional | Noh 2021; Sato 2025 | Fear of falling is elevated in prosthesis users because of device-confidence deficits (socket instability, foot slip, microprocessor-knee hesitation) that are partially addressable through fitting. | Phenotype-stratified SHAP (Sato 2025) showed fear of falling dominant in cautious walkers; in P&O users this cautious phenotype may reflect device distrust rather than intrinsic anxiety. | Tracking FES-I SHAP weight alongside device-confidence questionnaires during a fitting episode quantifies whether fitting changes translate into reduced fear-mediated fall risk. |
